# Identification of candidate genes and molecular markers for heat-induced brown discoloration of seed coats in cowpea [Vigna unguiculata (L.) Walp]

**DOI:** 10.1186/1471-2164-15-328

**Published:** 2014-05-01

**Authors:** Marti Pottorff, Philip A Roberts, Timothy J Close, Stefano Lonardi, Steve Wanamaker, Jeffrey D Ehlers

**Affiliations:** Department of Botany & Plant Sciences, University of California Riverside, Riverside, CA USA; Department of Nematology, University of California Riverside, Riverside, CA USA; Department of Computer Science and Engineering, University of California Riverside, Riverside, CA USA; Bill & Melinda Gates Foundation, Seattle, Washington USA

**Keywords:** Cowpea, Synteny, Legumes, Genomics, Marker-assisted selection, Heat-stress, Seed coat discoloration, Candidate genes, Ethylene

## Abstract

**Background:**

Heat-induced browning (*Hbs*) of seed coats is caused by high temperatures which discolors the seed coats of many legumes, affecting the visual appearance and quality of seeds. The genetic determinants underlying *Hbs* in cowpea are unknown.

**Results:**

We identified three QTL associated with the heat-induced browning of seed coats trait, *Hbs-1*, *Hbs-2* and *Hbs-3*, using cowpea RIL populations IT93K-503-1 (*Hbs* positive) x CB46 (*hbs* negative) and IT84S-2246 (*Hbs* positive) x TVu14676 (*hbs* negative). *Hbs-1* was identified in both populations, accounting for 28.3% -77.3% of the phenotypic variation. SNP markers 1_0032 and 1_1128 co-segregated with the trait. Within the syntenic regions of *Hbs-1* in soybean, Medicago and common bean, several ethylene forming enzymes, ethylene responsive element binding factors and an ACC oxidase 2 were observed. *Hbs-1* was identified in a BAC clone in contig 217 of the cowpea physical map, where ethylene forming enzymes were present. *Hbs-2* was identified in the IT93K-503-1 x CB46 population and accounted for of 9.5 to 12.3% of the phenotypic variance. *Hbs-3* was identified in the IT84S-2246 x TVu14676 population and accounted for 6.2 to 6.8% of the phenotypic variance. SNP marker 1_0640 co-segregated with the heat-induced browning phenotype. *Hbs-3* was positioned on BAC clones in contig512 of the cowpea physical map, where several ACC synthase 1 genes were present.

**Conclusion:**

The identification of loci determining heat-induced browning of seed coats and co-segregating molecular markers will enable transfer of *hbs* alleles into cowpea varieties, contributing to higher quality seeds.

**Electronic supplementary material:**

The online version of this article (doi:10.1186/1471-2164-15-328) contains supplementary material, which is available to authorized users.

## Background

Heat-induced browning is a phenomenon caused by high temperatures which discolor the seed coats of many legumes. The brown discoloration affects the visual appearance and quality of seeds which reduces its commercial value. The heat-induced brown discoloration affects the seed coats of soybean [1], common bean [2–4], cowpea [5], faba bean [6] and lentil [7]. The genetic determinants underlying heat-induced brown discoloration of seed coats in cowpea as well as other legumes is currently unknown.

In cowpea, the brown discoloration only appears on the seed coat and does not affect the underlying cotyledons [5]. In general, heat-induced browning can appear as patches or blotches, at the ends of the seed or over the entire surface of the cowpea seed [5]. Hall and Patel (1988) using microscopic and visual inspection noted that the tissue in the center of the brown discoloration appeared to be sunken and the seeds that were affected had rough textured seed coats. The brown discoloration of the seed coat has been observed in green seeds of fully matured cowpea pods, however, the brown discoloration is more distinct on dry matured seeds [5].

Hall and Patel (1985) studied the genetic inheritance of heat-induced browning in three cowpea populations derived from crosses with the heat-induced browning genotype, Tvu 4552; Tvu 4552 x CB5, Tvu 4552 x Bambey 21 and Tvu 4552 x PI 204647. It was confirmed with backcross populations that the *Hbs* trait is controlled by a single nuclear gene dominant to normal non-browning seed coat phenotype (*hbs*) [5]. The rate of the brown discoloration can be temperature controlled [5, 8]. An increase in night temperatures especially during pod-filling produced more browning of seeds with rough textured seed coats [8].

Molecular genetic tools and genomic resources have been developed for cowpea with an objective of enhancing breeding programs for the improvement of cowpea varieties for the United States, India, Brazil and numerous countries in Africa and Asia. These genomic resources have been integrated by using a 1536-SNP genotyping platform and include an EST-derived SNP consensus genetic map [9–11], known syntenic relationships between cowpea, *Medicago truncatula*, *Glycine max, Phaseolus vulgaris* and *Arabidopsis thaliana*, and a cowpea EST sequence collection housed in HarvEST:Cowpea database [12]. The cowpea physical map has been anchored to the consensus genetic map using the same SNP genotyping platform and sequenced BAC clones [13]. In addition, more than 500 diverse cowpea accessions have been SNP genotyped and a first draft of the cowpea genome sequence has been assembled [14]. These resources will enable dissection of underlying genetic components of target agronomic traits using genetic and physical mapping.

In this study, we identified three QTL, *Hbs-1*, *Hbs-2* and *Hbs-3*, associated with heat-induced browning of seed coats using the cowpea RIL populations IT93K-503-1 x CB46 and IT84S-2246 x TVu14676. SNP markers were identified which co-segregated with the heat-induced browning of seed coats phenotype in the *Hbs-1* and *Hbs-3* loci, and could be used for indirect selection in breeding a higher quality cowpea grain. Additionally, ethylene forming enzymes were identified as a cowpea candidate gene for the *Hbs-1* locus and an ACC synthase 1 gene was identified as a cowpea candidate gene for the *Hbs-3* locus.

## Results

### QTL analysis

**Figure 1:**
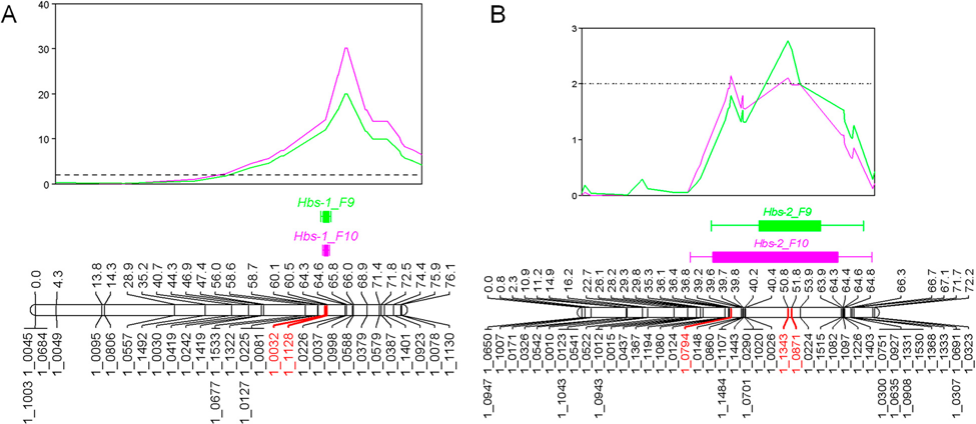
**A.** ***Hbs-1*** **in the IT93K-503-1 x CB46 population.** The heat-induced browning of seed coats locus, *Hbs-1*, was identified using datasets from two experiments. *Hbs-1 s*panned 60.09 cM to 60.53 cM on linkage group 8. SNP markers 1_0032 and 1_1128 were the most significant markers in the locus and are highlighted in red on the linkage group. However, 1_1128 had the highest association with the heat-induced browning phenotype, accounting for 62.8% and 77.3% of the phenotypic variance. The significance threshold is indicated by the horizontal broken line. **B**. *Hbs-2* in the IT93K-503-1 x CB46 population. The heat-induced browning of seed coats locus, *Hbs-2*, was identified using datasets from two experiments. *Hbs-2 s*panned 36.82 cM to 51.79 cM on linkage group 3. SNP marker 1_1342 accounted for the highest percent phenotypic variance of 9.5% (LOD 2.11) and 12.3% (LOD 2.77) and is highlighted in red on the linkage group. The significance threshold is indicated by the horizontal broken line.

QTL analysis of two phenotypic datasets for the IT93K-503-1 (*Hbs*) x CB46 (*hbs*) population identified two loci for the heat-induced browning phenotype. We designated the major locus as *Hbs-1* and the minor locus as *Hbs-2. Hbs-1* spanned 35.21 cM to 76.13 cM on linkage group 8 of the IT93K-503-1 x CB46 individual genetic map (Figure 1a, Additional file 1). The most significant region (2-LOD) spanned 60.09 cM to 60.53 cM (0.44 cM total length) (Figure 1A, Additional file 1). SNP markers 1_0032 and 1_1128 were the most significant markers for both experiments (Additional file 1). Marker 1_1128 had the highest association with the heat-induced browning phenotype and accounted for 62.8% (LOD score 20.01) and 77.3% (LOD score 30.19) of the phenotypic variance in the two experiments, respectively (Additional file 1). The corresponding *Hbs-1* locus was positioned on the cowpea consensus genetic map where it spanned 25.14 cM to 57.58 cM on linkage group 5; the most significant region (2-LOD) spanned from 45.27 cM to 45.76 cM (Additional file 2 and Additional file 3). The minor locus, *Hbs-2*, spanned from 36.82 cM to 51.79 cM on linkage group 3 of the individual map (Figure 1B, Additional file 4). SNP marker 1_1342 accounted for the highest percent phenotypic variance of 9.5% (LOD 2.11) and 12.3% (LOD 2.77) (Additional file 4). *Hbs-2* was positioned on the cowpea consensus genetic map where it spanned from 31.28 cM to 58.09 cM on LG6 (Additional file 2 and Additional file 5). *Hbs-2* overlapped SNP markers 1_1346 (55.50 cM) and 1_0437 (57.41 cM) which were previously identified within the stage II heat-tolerance QTL *Cht-3*[15]. Heat shock proteins (HSP), DNA J heat shock family protein (DNA J HSP) and hydroxyproline-rich glycoprotein family (HPR) were identified as potential candidate genes within the syntenic loci of *Cht-3* in soybean [15]. The overlap of the *Hbs-2* locus with the *Cht-3* locus does not concur with the findings of Hall and Patel (1988) in which the heat-induced browning of seed coats trait was not linked with heat tolerance in early floral bud development. However, this QTL mapping study and Lucas et al. (2013) used different cowpea populations than the study by Hall and Patel (1988).

**Figure 2:**
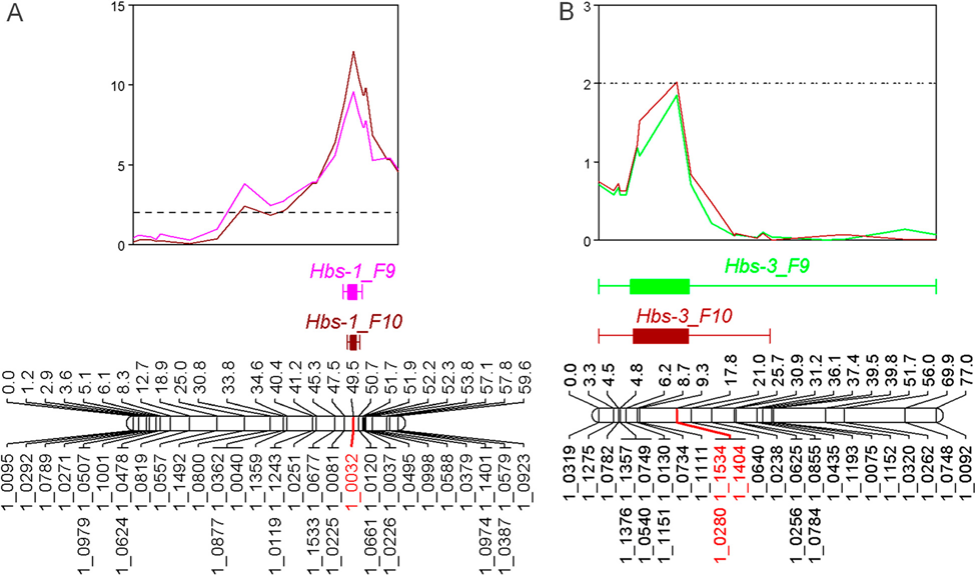
**A.** ***Hbs-1*** **in the IT84S-2246 x TVu14676 population.** The major locus for the heat-induced browning of seed coats phenotype was mapped using datasets from two experiments. *Hbs-1* spanned 49.51 cM to 50.69 cM on linkage group 9. SNP marker 1_0032 was the most significant marker for both experiments accounting for 28.3% and 34.1% of the phenotypic variance and is highlighted in red on the linkage group. The significance threshold is indicated by the horizontal broken line. **B**. *Hbs-3* in the IT84S-2246 x TVu14676 population. The minor locus for the heat-induced browning phenotype was mapped using two experimental datasets (Interval Mapping analysis shown). *Hbs-2* spanned 8.67 cM to 20.97 cM on linkage group 3. SNP markers 1_0280, 1_1534 and 1_1404 shared the same marker bin, accounting for 6.2% and 6.8% of the phenotypic variance and are highlighted in red on the linkage group. The significance threshold is indicated by the horizontal broken line on the graph.

The heat-induced browning of seed coats trait was mapped in the IT84S-2246 (*Hbs*) x TVu14676 (*hbs*) population using two phenotypic datasets, wherein one major and one minor QTL were identified. The major locus was found to overlap directly with the IT93K-503-1 x CB46 *Hbs-1* locus on the cowpea consensus genetic map, spanning from 34.66 cM to 56.58 cM on linkage group 5 (Additional file 2 and Additional file 3). The most significant region (2-LOD) spanned from 45.27 cM to 46.51 cM (Additional file 2 and Additional file 3). Therefore, the major heat-induced browning locus identified in the IT84S-2246 x TVu14676 population will also be referred as *Hbs-1. Hbs-1* spanned 24.98 cM to 59.60 cM on linkage group 9 of the IT84S-2246 x TVu14676 individual map (Figure 2A, Additional file 6). SNP 1_0032 was the most significant marker for both experiments, accounting for 28.3% and 34.1% of the phenotypic variance and LOD scores of 9.54 and 12.05, respectively (Additional file 6). The minor locus which spanned 17.79 cM to 20.97 cM on linkage group 3 was designated as *Hbs-3* (Figure 2B, Additional file 7). SNP markers 1_0280, 1_1534 and 1_1404 shared the same marker bin and accounted for 6.2% and 6.8% of the phenotypic variance with LOD scores of 1.85 and 2.02, respectively (Additional file 7). The *Hbs-3* locus was positioned on the cowpea consensus genetic map where it spanned 36.0 cM to 37.96 cM on linkage group 1 (Additional file 2 and Additional file 8).

### Marker-trait association within the *Hbs-1* and *Hbs-3* loci

**Figure 3:**
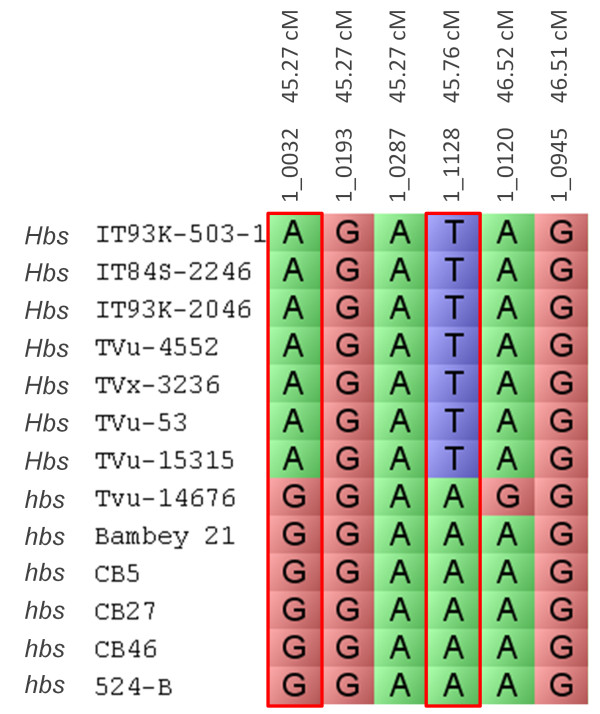
**Marker-trait association of the** ***Hbs-1*** **locus.** A marker-trait association of the *Hbs-1* locus was analyzed using thirteen cowpea genotypes which differ in their response to heat-induced browning of seed coats phenotype. IT93K-503-1, IT84S-2246, IT93K-2046, TVu-4552 and TVx-3236, TVu-53 and TVu-15315 were positive for the heat-induced browning phenotype and are referred to as *Hbs*. TVu-14676, CB5, CB27, CB46, 524B and Bambey 21 are negative for the heat-induced browning phenotype and are referred to as *hbs*. The most significant region of the *Hbs-1* locus spanned from 45.27 cM to 47.18 cM on the cowpea consensus genetic map linkage group 5. SNP markers 1_0032 and 1_1128 alleles co-segregated with the positive (*Hbs*) and negative (*hbs*) genotypes. The *Hbs-1* positive genotypes were associated with the adenine nucleotide (color-coded green) at the 1_0032 locus, while *hbs-1* negative genotypes were associated with the guanine nucleotide (color-coded red). The adenine/guanine SNP in marker 1_0032 is position 469 of cowpea unigene 5294 which was annotated as an AMP dependant ligase/synthetase and can be viewed in HarvEST:Cowpea. At the 1_1128 locus, the *Hbs-1* positive genotypes were associated with the thymine nucleotide (color-coded blue) while the *hbs-1*-negative genotypes were associated with the adenine nucleotide (color-coded green). The thymine/adenine SNP in marker 1_1128 is position 950 of cowpea unigene 4874, which was annotated as an ubiquitin-protein ligase and can be viewed in HarvEST: Cowpea.

Cowpea genotypes which differ in their phenotype to heat-induced browning of seed coats were chosen for a marker-trait association to narrow the *Hbs-1* and *Hbs-3* loci. IT93K-503-1, IT84S-2246, IT93K-2046, TVu-4552, TVx-3236, TVu-53 and TVu-15315 were positive for the heat-induced browning phenotype and are referred to as *Hbs* (Additional file 9). TVu-14676, CB5, CB27, CB46, 524B and Bambey 21 were negative for the heat-induced browning phenotype and are referred to as *hbs* (Additional file 9). Within the most significant region of the *Hbs-1* locus, which extended from 45.27 cM to 46.51 cM on LG5, 2 out of 6 SNP markers co-segregated with *Hbs* (positive) and *hbs* (negative) genotypes (Figure 3). The *Hbs* positive genotypes had the adenine nucleotide at the 1_0032 locus, while the *hbs* negative genotypes were associated with the guanine nucleotide (Figure 3). The adenine/guanine SNP in marker 1_0032 is at position 469 of cowpea unigene 5294 and can be viewed in HarvEST:Cowpea [12]. For SNP marker 1_1128, the *Hbs* positive genotypes had the thymine nucleotide which is color-coded blue while the *hbs* negative genotypes had the adenine nucleotide and were color-coded green (Figure 3). The thymine/adenine SNP in marker 1_1128 is position 950 of cowpea unigene 4874 and can be viewed in HarvEST:Cowpea [12]. SNP markers 1_0032 and 1_1128 could both be used as molecular markers to screen against the heat-induced browning of seed coats trait in cowpea.

**Figure 4:**
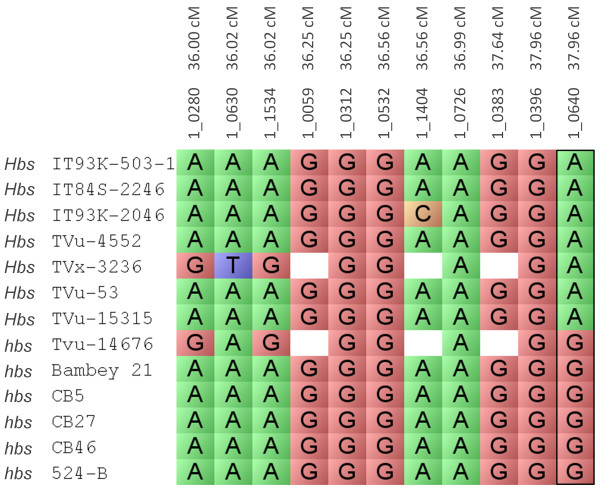
**Marker-trait association of the** ***Hbs-3*** **locus.** A marker-trait association of the *Hbs-3* locus was analyzed using thirteen cowpea genotypes which differ in their response to heat-induced browning of seed coats phenotype. IT93K-503-1, IT84S-2246, IT93K-2046, TVu-4552 and TVx-3236, TVu-53 and TVu-15315 were positive for the heat-induced browning phenotype and are referred to as *Hbs*. TVu-14676, CB5, CB27, CB46, 524B and Bambey 21 are negative for the heat-induced browning phenotype and are referred to as *hbs*. The *Hbs-3* locus spanned from 36.00 cM to 37.96 cM on the cowpea consensus genetic map linkage group 1. SNP marker 1_0640 alleles co-segregated with the positive (*Hbs*) and negative (*hbs*) genotypes. The *Hbs-3* positive genotypes were associated with the adenine nucleotide (color-coded green) while the *hbs-3* negative genotypes were associated with the guanine nucleotide (color-coded red). The adenine/guanine SNP in marker 1_0640 is position 348 of cowpea unigene 2077, which was annotated as a 60S ribosomal protein and can be viewed in HarvEST:Cowpea.

The same *Hbs* positive and *hbs* negative genotypes were used to narrow the *Hbs-3* locus, which spanned 36.00 cM to 37.96 cM on LG1. The alleles for SNP marker 1_0640 co-segregated with *Hbs* positive and *hbs* negative phenotypes (Figure 4). The *Hbs* positive genotypes were associated with the adenine nucleotide while *hbs* negative genotypes were associated with the guanine nucleotide (Figure 4). The SNP for marker 1_0640 is position 348 and can be viewed in HarvEST: Cowpea [12]. SNP marker 1_0640 could also be used for screening germplasm and breeding material against the minor heat-induced seed coat browning locus.

Theoretically, these three “tagged SNPs” could be used to genotype the *Hbs-1* and *Hbs-3* haplotype blocks to determine the phenotype, rather than the sixty-six SNP markers within the *Hbs-1* QTL and eleven SNP markers within the *Hbs-3* QTL. However, a larger and more diverse set of cowpea germplasm would be needed to test and validate this approach.

### Synteny of *Hbs-1* with soybean, Medicago and common bean

The *Hbs-1* locus was examined for synteny with the soybean genome and a high co-linearity was observed for soybean chromosomes 2 and 14 (Additional file 10a). Soybean orthologs for eleven out of twenty-three SNP markers were identified in the co-linear region of soybean chromosome 2, spanning from Glyma02g42560 to Glyma02g43640, which corresponded to 44.42 cM to 46.51 cM of the *Hbs-1* locus (Additional file 11). The region surrounding the soybean orthologs to SNP markers 1_1128 and 1_0032 was examined on the soybean genome browser on the Phytozome website [16] for genes associated with heat stress. Several soybean loci were closely linked with the soybean ortholog for cowpea SNP 1_1128 and were considered candidate genes for the heat-induced browning of seed coats phenotype; Glyma02g43500 was annotated as an ATERF3/ERF3 (ethylene responsive element binding factor) and Glyma02g43560, Glyma02g43580 and Glyma02g43600 were annotated as ethylene-forming enzymes (EFE) (Additional file 11). The syntenic locus on soybean chromosome 14 spanned from Glyma14g05250 to Glyma14g06330 which corresponded to 44.42 cM to 47.18 cM of the *Hbs-1* locus on the cowpea consensus genetic map (Additional file 11). Soybean locus Glyma14g05470 was annotated as an ATERF3/ERF3 (ethylene responsive element binding factor 3) and was considered a putative candidate gene for the *Hbs-1* locus (Additional file 11). Other putative soybean candidate genes for the *Hbs-1* locus included Glyma14g05350, Glyma14g05360 and Glyma14g05390, which were annotated as EFEs (Additional file 11).

The *Hbs-1* locus was syntenic with *M. truncatula* chromosome 5 where it spanned from Medicago locus Medtr5g018870 to Medtr5g093060, which corresponded to 44.42 cM to 47.18 cM of the *Hbs-1* locus on the cowpea consensus genetic map (Additional file 10b and Additional file 12). Several Medicago genes were observed in the region of Medicago orthologs to SNPs 1_0032 and 1_1128 and were considered as putative candidate genes for the *Hbs-1* locus; Medtr5g092410, Medtr5g092450 and Medtr5g092470 were annotated as ethylene response factor 3 (ERF3), Medtr5g092480 was annotated as ERF11 and Medtr5g092760 was annotated as an EFE (Additional file 12).

SNP markers for the *Hbs-1* locus were examined in the common bean genome to determine if a syntenic relationship existed; the *Hbs-1* locus was highly co-linear with common bean chromosome 8, extending from locus Phvul.008G213300 to Phvul.008G214300 (Additional file 13 and Additional file 14). The marker-order between cowpea and common bean was conserved, although the gene order was inverted (Additional file 14). Phvul.008G213800 and Phvul.008G213900 were slightly upstream from co-segregating SNP marker 1_1128 and were annotated as ethylene-forming enzyme and ACC oxidase 2 (Additional file 14).

### *Hbs-1*, *Hbs-2* and *Hbs-3* on the cowpea physical map

The cowpea physical map [13] which has been anchored to the cowpea consensus genetic map via SNP markers and sequenced BAC clones was used to identify a contig which overlapped the *Hbs-1, Hbs-2* and *Hbs-3* loci. Significant markers from the QTL analysis and closely linked markers from the cowpea consensus genetic map identified cowpea BAC contigs and clones which overlapped the heat-induced browning QTLs. SNP markers 1_1128 and 1_0120 which were identified in BAC clone CM018C23 of contig217 positioned the *Hbs-1* locus on the physical map (Additional file 3). BAC clone CM018C23 has 84 fingerprint bands which estimated its size as 137,760 bps [13]. Annotations for BAC clone CM018C23 revealed the presence of three ethylene-forming enzymes (EFE) (Table 1). The fact that EFEs and other genes involved in the biosynthesis of ethylene were identified in the syntenic loci of *Hbs-1* in cowpea, soybean, Medicago and common bean reinforces the utility of syntenic relationships in identifying candidate genes between the legume species.

**Table 1:** **Annotations for the** ***Hbs-1*** **locus on cowpea BAC clone CM018C23 of contig217 of the cowpea physical map**

BAC clone/NODE	e-score	***P. vulgaris*** locus/cowpea SNP	Annotation
CM018C23_VU2.3_NODE_0001	2.00E-69	Phvul.008G214100.1	Ankyrin repeat family protein
CM018C23_VU2.3_NODE_0002	0	Phvul.008G214000.1	Ankyrin repeat family protein
CM018C23_VU2.3_NODE_0003	1.00E-142	Phvul.008G213700.1	Calcineurin-like metallo-phosphoesterase superfamily protein
CM018C23_VU2.3_NODE_0004	3.00E-79	Phvul.008G214300.1/1_1128	Phosphotyrosine protein phosphatases superfamily protein
CM018C23_VU2.3_NODE_0011	3.00E-143	Phvul.008G214000.1	Ankyrin repeat family protein
CM018C23_VU2.3_NODE_0013	8.00E-124	Phvul.007G135600.1	Ethylene-forming enzyme
CM018C23_VU2.3_NODE_0021	8.00E-92	Phvul.008G214000.1	Ankyrin repeat family protein
CM018C23_VU2.3_NODE_0022	1.00E-47	Phvul.008G214000.1	Ankyrin repeat family protein
CM018C23_VU2.3_NODE_0024	2.00E-151	Phvul.008G214200.1/1_0120	Ethylene-forming enzyme
CM018C23_VU2.3_NODE_0030	6.00E-147	Phvul.008G213800.1	Ethylene-forming enzyme

The length of the *Hbs-2* QTL was quite extensive and many contigs overlapped the region. The most significant marker from the QTL analysis, SNP 1_1343 was imbedded in BAC clone CH082B14 of contig606 (Additional file 5). However, no genes of interest were identified in the clone.

The *Hbs-3* locus was also positioned on the cowpea physical map where BAC contigs 674, 512 and 661 incompletely spanned the *Hbs-3* locus (Additional file 8). SNP marker 1_0640 which co-segregated with the heat-induced browning phenotype in the *Hbs-3* locus was not identified in the cowpea physical map. However, SNP 1_0640 (37.96 cM) shared the same marker bin as SNP 1_0396 (37.96 cM) on the cowpea consensus genetic map, so it was considered the closest marker to the *Hbs-3* locus (Additional file 8). SNP 1_0396 was identified in BAC clone CM042F21 of contig 512, but no genes associated with heat stress were found in the annotations. SNP marker 1_0383 (37.64 cM) which is 0.32 cM away from SNP 1_0640 (37.96 cM) was identified in BAC clones CH047M01 and CM014K16 which are also contained within contig512 (Additional file 8). Annotations for CH047M01and CM014K16 revealed the presence of two 1-aminocyclopropane-1-carboxylate (ACC) synthase 1 genes as possible candidate genes for the *Hbs-3* locus (Table 2).

**Table 2:** **Annotations for the** ***Hbs-3*** **locus on cowpea BAC clone CM042F21, CH047M01 and CM014K16 of contig512 of the cowpea physical map**

BAC clone/NODE	e-score	***P. vulgaris*** locus/cowpea SNP	***P. vulgaris*** gene model
CM042F21_VU2.3_NODE_0001	0	Phvul.008G060300.1	Brassinosteroid-responsive RING-H2
CM042F21_VU2.3_NODE_0002	0	Phvul.008G059900.1	Protein of unknown function
CM042F21_VU2.3_NODE_0004	0	Phvul.008G060100.1	Ferric reduction oxidase 2
CM042F21_VU2.3_NODE_0005	0	Phvul.008G059800.1	Plant U-Box 15
CM042F21_VU2.3_NODE_0007	5.00E-84	Phvul.008G060500.1	Transmembrane protein-related
CM042F21_VU2.3_NODE_0008	9.00E-146	Phvul.008G059200.1/1_0383	Ribosomal L5P family protein
CM042F21_VU2.3_NODE_0009	3.00E-67	Phvul.008G059300.1	Peptidase family M48 family protein
CM042F21_VU2.3_NODE_0010	0	Phvul.008G059400.1	Protein kinase superfamily protein
CM042F21_VU2.3_NODE_0016	0	Phvul.008G060000.1	Fringe-related protein
CM042F21_VU2.3_NODE_0023	0	Phvul.008G059500.1/1_0396	Protein of unknown function
CH047M01_VU1.3_NODE_0002	7.00E-140	Phvul.008G058200.1	Alpha/beta-Hydrolases superfamily protein
CH047M01_VU1.3_NODE_0003	0	Phvul.008G058300.1	WRKY family transcription factor
CH047M01_VU1.3_NODE_0007	0	Phvul.002G179300.1	Polynucleotidyl transferase
CH047M01_VU1.3_NODE_0015	0	Phvul.008G058400.1	ACC synthase 1
CH047M01_VU1.3_NODE_0019	5.00E-131	Phvul.008G058700.1	Cysteine-rich RLK (RECEPTOR-like protein kinase) 29
CM014K16_VU2.3_NODE_0003	0	Phvul.008G058500.1	Heavy metal transport/detoxification superfamily protein
CM014K16_VU2.3_NODE_0012	4.00E-140	Phvul.008G058200.1	Alpha/beta-Hydrolases superfamily protein
CM014K16_VU2.3_NODE_0014	0	Phvul.008G058400.1	ACC synthase 1
CM014K16_VU2.3_NODE_0016	0	Phvul.008G059400.1	Protein kinase superfamily protein
CM014K16_VU2.3_NODE_0019	0	Phvul.008G058600.1	Cysteine-rich RLK (RECEPTOR-like protein kinase) 29
CM014K16_VU2.3_NODE_0019	7.00E-24	Phvul.007G052500.1	Cysteine-rich RLK (RECEPTOR-like protein kinase) 29
CM014K16_VU2.3_NODE_0022	1.00E-82	Phvul.008G058000.1	WRKY DNA-binding protein 24
CM014K16_VU2.3_NODE_0023	0	Phvul.008G058300.1	WRKY family transcription factor
CM014K16_VU2.3_NODE_0025	6.00E-33	Phvul.008G059400.1	Protein kinase superfamily protein
CM014K16_VU2.3_NODE_0027	0	Phvul.008G059100.1/1_0383	Protein of unknown function
CM014K16_VU2.3_NODE_0058	3.00E-67	Phvul.008G059300.1	Peptidase family M48 family protein
CM014K16_VU2.3_NODE_0059	0	Phvul.008G058900.1	NAD(P)-binding Rossmann-fold superfamily protein
CM014K16_VU2.3_NODE_0003	0	Phvul.008G058500.1	Heavy metal transport/detoxification superfamily protein
CM014K16_VU2.3_NODE_0012	4.00E-140	Phvul.008G058200.1	Alpha/beta-Hydrolases superfamily protein
CM014K16_VU2.3_NODE_0014	0	Phvul.008G058400.1	ACC synthase 1
CM014K16_VU2.3_NODE_0016	0	Phvul.008G059400.1	Protein kinase superfamily protein
CM014K16_VU2.3_NODE_0019	0	Phvul.008G058600.1	Cysteine-rich RLK (RECEPTOR-like protein kinase) 29
CM014K16_VU2.3_NODE_0019	7.00E-24	Phvul.007G052500.1	Cysteine-rich RLK (RECEPTOR-like protein kinase) 29
CM014K16_VU2.3_NODE_0022	1.00E-82	Phvul.008G058000.1	WRKY DNA-binding protein 24

## Discussion

### Candidate genes for the *Hbs-1* and *Hbs-3* loci

Synteny between cowpea, soybean, Medicago and common bean as well as the integrated genomic resources for cowpea were used to identify ethylene forming enzymes (EFE) as the cowpea candidate gene for the *Hbs-1* locus and an ACC synthase 1 gene for the *Hbs-3* locus. The plant hormone ethylene has long been associated with plants’ ability to systematically relay information regarding abiotic and biotic stress. The biosynthesis of ethylene is dependent on the rate-limiting step; conversion of S-adenosylmethionine (AdoMet)(SAM) to ACC by the enzyme (ACC) synthase (ACS) [17]. Thus, ACS is considered to be the most important enzyme in this pathway. EFEs are involved in the secondary reaction forming ethylene; oxidation of ACC to ethylene [18, 19].

The importance of ethylene closely associated with heat stress has been indicated by several studies. Researchers examining heat-induced oxidative damage in *Arabidopsis* showed that ethylene, abscisic acid (ABA) and salicylic acid (SA) were key to protecting against heat-induced stress; an ethylene-insensitive mutant *ethylene resistant 1* (*etr-1*) showed an increase of susceptibility to heat [20]. In another study, *Arabidopsis* insensitive mutants to ethylene signaling, *etr-1* and *ethylene insensitive 2* (*ein2*), showed a significant reduction in tolerance to basal-thermotolerance compared to wild-type [21]. Munne-Bosch et al. (2004) sought to determine if airborne ethylene such as found in highly polluted areas affected plant stress tolerance. They observed that ethylene-fumigated holm oak trees showed much less tolerance to heat stress and heat stress combined with drought stress than controls [22]. Ethylene treated oak trees showed oxidative stress at 35°C whereas the controls showed a heat tolerance up to 50°C [22]. Additionally, ethylene treated trees showed more visual leaf area damage than controls [22]. Investigators of a heat-susceptible hard red winter wheat found that there was a 6-fold increase of ethylene in wheat kernels vs. no change in a heat-tolerant wheat cultivar ‘Halberd’ [23]. Similarly, a 7-fold increase of ethylene was produced in embryos and a 12-fold increase of ethylene was found in the flag leaf of the heat-susceptible wheat genotype [23]. The fact that ethylene is involved in heat stress regulation in many plant species makes ACC synthase and EFEs plausible candidate genes for regulating heat-induced browning of seed coats in cowpea. It is interesting to note that the two candidate genes for heat-induced brown discoloration in cowpea are two enzymes intricately involved in the ethylene biosynthesis pathway.

## Conclusion

In this study, we report the identity of three loci, *Hbs-1, Hbs-2 and Hbs-3*, associated with heat-induced browning of seed coats in cowpea. The major heat-induced browning locus, *Hbs-1*, was observed in both RIL populations, IT93K-503-1 x CB46 and IT84S-2246 x TVu14676. *Hbs-2* was a minor locus identified in the IT93K-503-1 x CB46 population, while *Hbs-3* was a minor locus observed in the IT84S-2246 x TVu14676 population. Parental lines IT93K-503-1 and IT84S-2246 both exhibited the heat-induced browning of seed coats trait. The genetic and physical mapping and identity of candidate genes for the *Hbs-1* and *Hbs-3* loci were conducted utilizing integrated cowpea consensus genetic and physical maps as well as syntenic relationships with soybean, Medicago and common bean. The major locus, *Hbs-1*, was narrowed to cowpea BAC clone CM018C23 of BAC contig 217 on the cowpea physical map, where ethylene-forming enzymes (EFE) were present and considered as putative cowpea candidate genes. The minor locus, *Hbs-3* was positioned on BAC clones CM042F21, CH047M01 and CM014K16 of contig512 of the cowpea physical map where ACC synthase 1 genes were present and considered as potential candidate genes.

The practical outcome of this study was the identification of molecular markers 1_0032 and 1_1128 co-segregating with the *Hbs-1* phenotype and SNP marker 1_0640 which co-segregated with the *Hbs-3* phenotype. The heat-induced browning phenotype is present in about 20% of the elite IITA breeding lines. Since the *Hbs* phenotype is not manifested unless the breeding material is exposed to the appropriate heat conditions, having markers to screen against the trait at the seedling stage would limit the number of plants needed to be grown to maturity. This would enable an efficient selection process to ensure that cowpea cultivars being bred do not carry the heat-induced browning trait. These approaches should expedite variety development by at least halving the current traditional breeding selection process which relies on time-consuming and costly phenotyping under heat stress conditions. Future goals include functional analysis of cowpea *Hbs-1* and *Hbs-3* candidate genes, which enhance our understanding of the heat-induced browning phenomenon as well as provide a ‘perfect marker’ which would further improve marker-assisted breeding efficiency.

## Methods

### Plant populations

The IT93K-503-1 (*Hbs*) x California Blackeye ‘46’ (*hbs*) population consisted of 113 lines which were advanced to the F_10_ generation using single seed decent. IT93K-503-1 is an advanced breeding line developed by International Institute for Tropical Agriculture (IITA) which features several important traits, including drought tolerance [24], resistance to *Fusarium oxysporum* f.sp. *tracheiphilum* (Fot) race 3 [25] and 4 [26], and resistance to *Macrophomina phaseolina*[27]. California Blackeye 46 is a California bred variety from University of California, Davis and has important qualities such as resistance to Fot race 3 [28]. The F_9_ and F_10_ generation were phenotyped for the heat-induced browning trait using a set of 99 RILs.

The IT84S-2246 (*Hbs*) x TVu14676 (*hbs*) consisted of 136 RILs which were advanced to the F_8_ generation using single seed descent. IT84S-2246 is an IITA breeding line which has strong resistance to several root-knot nematodes including *Rk*-virulent *M.incognita* and *Rk*-aggressive *M. javanica*[29], aphids, bruchids and thrips and several other diseases [30]. TVu14676 is a cowpea cultivar developed by IITA and is resistant to the parasitic plant *Striga gesnerioides* races SGl, SG2, SG3 and SG5 [31]. The F_9_ and F_10_ generation were phenotyped for the heat-induced browning of seed coats trait using 131 and 134 out of 146 RILs. All cowpea materials were available from the University of California Riverside cowpea germplasm collection.

### Experiments and phenotyping

Greenhouse experiments were conducted at the University of California Riverside, Citrus Research Station. Two independent experiments for each population were conducted to phenotype for the heat-induced browning of seed coats trait. Seeds of parents and RILs were planted into separate 18.93 L pots filled with University of California Soil Mix II [32] and watered daily. Greenhouse day temperatures varied with the mean daily maximum of 35°C and a mean nightly minimum of 24°C. The seeds were harvested when mature after the pods had dried.

Heat-induced browning was phenotyped by a visual inspection of dried seeds obtained from mature plants exposed to high temperatures under greenhouse conditions. Brown discoloration covering the entire surface of the seed or in smaller patches was considered positive for the heat-induced browning trait and were recorded as a “1” (Additional file 15). Seeds which did not display brown discoloration at all were considered negative for the trait and were recorded as a “0” (Additional file 15).

### SNP genotyping

The IT93K 503–1 x CB46 and IT84S-2246 x TVu14676 populations were genotyped at the F_8_ generation using bi-allelic SNP markers using the 1536 Illumina Golden Gate Assay as previously described in Muchero et al. (2009). The cowpea cultivars used for the marker-trait association study were SNP genotyped at the F_8_ or higher generation.

### Genetic maps

The genetic map for the IT93K 503–1 x CB46 RIL population was previously created and is included in the cowpea consensus genetic map vs.2 [9], vs.3 [10], and vs.4 [11]. The individual map was generated using 114 RILs and 374 SNP markers and consisted of seventeen linkage groups which spanned approximately 639.6 cM [11]. The genetic map for IT84S-2246 x TVu14676 was also previously included in the vs.2, vs.3, and vs.4 maps. The individual map was generated using 136 RILs and 345 SNP markers and consists of fourteen linkage groups which span approximately 666.9 cM [11]. The cowpea consensus genetic map vs. 4 [11] was used for this study and is an updated version of the vs.2 and vs.3 maps. The vs. 4 consensus map consisted of ten RIL populations and two breeding populations which increased the marker density and improved the marker order [11]. The map is 680 cM in length and contains 1107 markers with an average of 0.65 cM between markers [11]. The current SNP-based cowpea linkage map is included in a publicly available browser called HarvEST:Cowpea [12].

### Statistical analyses

Kruskal-Wallis and Interval Mapping analysis packages of MapQTL 5.0 software were used to conduct the QTL analysis [33]. A QTL was considered significant if the same QTL was identified using both phenotypic datasets and if the statistical tests for the markers met significance thresholds for both Kruskal-Wallis and Interval Mapping analyses. A significance threshold was set at 0.05 for Kruskal-Wallis analysis and LOD thresholds for the Interval Mapping analysis were calculated using 1000 permutations at the 0.05 significance level. A 95% confidence interval was used to determine the span of QTLs using 1-LOD and 2-LOD to determine left and right margins. QTLs were visualized using MapChart 2.2 software [34].

### Synteny

Synteny was examined between cowpea and *G. max*, cowpea and *M. truncatula* and cowpea and *P. vulgaris* using EST-derived SNP markers previously BLASTed and aligned to the sequenced genomes. Syntenic relationships between the different genomes can be examined in HarvEST:Cowpea database [12]. The soybean, Medicago and common bean annotations were taken from the Phytozome webpage [16]. Syntenic maps were drawn using HarvEST:Cowpea using a cut off e-score value of -10, with a minimum number of 10 lines drawn per linkage group [12]. Due to limited resolution in the software images, not all markers are presented in the screenshot images output from Harvest:Cowpea. The linkage group must be magnified using the HarvEST:Cowpea software in order to view each individual marker.

### Marker-trait association

Genotypic data comprised of cowpea varieties and their SNP call for each locus of the cowpea consensus genetic map were visualized using Flapjack software [35].

### Cowpea physical map

The physical map was developed using an advanced African breeding line IT97K-499-35 [13]. It consists of two BAC clone libraries developed using restriction enzymes *Hind*III and *Mbo*I (Amplicon Express, Pullman, WA). Contigs were assembled using the snapshot method of DNA fingerprinting [36] and assembly was completed at the University of California Davis by Ming Cheng Luo. The final physical map is an assembly of 43,717 BACs with an 11x genome depth of coverage [13]. The cowpea physical map manuscript is currently in preparation. The size of the BAC clones was estimated by multiplying the number of unique bands generated from the fingerprinting assay by 1640 bp (personal communication, MC Luo).

Sequences were generated for cowpea BACs using an Illumina HiSeq 2000 sequencer by John Weger at the Institute of Integrative Genomics Biology, University of California, Riverside (UCR) from DNA samples prepared by Yaqin Ma (UCR). Sequences of each BAC were generated from paired-end 100 base reads using the combinatorial pooling method described previously [37]. A NODE is defined as a sequence or contig which can be consistently reconstructed using the sequencing reads [38, 39]. All sequence data are publicly available via the Harvest:Cowpea database [12] and version 0.03 of the assembled cowpea genome [14]. Cowpea genome version 0.03 which contained approximately 200 Mb of assembled scaffolds and contigs covered about 97% of previously identified cowpea genes is available for BLAST searches and sequence retrieval [14].

## Electronic supplementary material

Additional file 1: **QTL analysis of** ***Hbs-1*** **in IT93K-503-1 x CB46 population.** (DOCX 16 KB)

Additional file 2: ***Hbs-1***, ***Hbs-2 and*** ***Hbs-3*** **on the cowpea consensus genetic map.** Heat-induced browning of seed coats QTLs were positioned on the cowpea consensus genetic map using SNP markers identified in the QTL analyses. *Hbs-1* and *Hbs-2* (labeled light blue) were identified in the IT93K-503-1 x CB46 population. *Hbs-1* and *Hbs-3* (labeled magenta) were identified in the IT84S-2246 x TVu14676 population. The most significant SNP marker for each QTL is highlighted in the corresponding color on the linkage group. SNP marker 1_0032 is labeled red since it was the most significant marker for both the *Hbs-1* locus identified in IT93K-503-1 x CB46 and the IT84S-2246 x TVu14676 population. (PDF 47 KB)

Additional file 3: ***Hbs-1*** **in IT93K-503-1 x CB46 and IT84S-2246 x TVu14676 individual maps, cowpea consensus genetic map, and the cowpea physical map.** (DOCX 14 KB)

Additional file 4: **QTL analysis of** ***Hbs-2*** **in IT93K-503-1 x CB46 population.** (DOCX 12 KB)

Additional file 5: ***Hbs-2*** **in IT93K-503-1 x CB46 genetic map, cowpea consensus genetic map, and the cowpea physical map.** (DOCX 16 KB)

Additional file 6: **QTL analysis of** ***Hbs-1*** **in the IT84S-2246 x TVu14676 population.** (DOCX 16 KB)

Additional file 7: **QTL analysis of** ***Hbs-3*** **in the IT84S-2246 x TVu14676 population.** (DOCX 12 KB)

Additional file 8: ***Hbs-3*** **in the IT84S-2246 x TVu14676 population, cowpea consensus genetic map and cowpea physical map.** (DOCX 14 KB)

Additional file 9: **Origins of genotypes used for the marker-trait association.** (DOCX 12 KB)

Additional file 10: **a. Synteny figure of** ***Hbs-1*** **locus with** ***G. max.*** Synteny was examined for the *Hbs-1* locus between cowpea and *G. max* using EST-derived SNP markers previously BLASTed and aligned to the sequenced genome. The *Hbs-1* locus which spanned 45.27 cM to 47.18 cM on the cowpea consensus genetic map linkage group 5 was determined to be syntenic with soybean chromosomes 2 and 14. The syntenic locus in soybean chromosome 2 extended from soybean locus Glyma02g42560 to Glyma02g43640 which corresponded to 44.42 cM to 46.51 cM of the *Hbs-1* locus. The syntenic locus on soybean chromosome 14 spanned from Glyma14g05250 to Glyma14g06330 which corresponded to 44.42 cM to 47.18 cM of the *Hbs-1* locus on the cowpea consensus genetic map. Ethylene responsive element binding factor 3 and 11 and ethylene forming enzymes were observed in the syntenic regions of soybean and were considered candidate genes for the *Hbs-1* locus. b. Synteny figure of *Hbs-1* locus with *M. truncatula*. Synteny was examined for the *Hbs-1* locus between cowpea and *M. truncatula* using EST-derived SNP markers previously BLASTed and aligned to the sequenced genome. The *Hbs-1* locus which spanned 45.27 cM to 47.18 cM on the cowpea consensus genetic map linkage group 5 was determined to be syntenic with *M. truncatula* chromosome 5 where it spanned from Medicago locus Medtr5g018870 to Medtr5g093060. Ethylene response factor 3 (ERF3) and an ethylene forming enzyme were present in the locus and were considered candidate genes. (TIFF 2 MB)

Additional file 11: **Synteny table of** ***Hbs-1*** **in** ***Glycine max*** **chromosomes 2 and 14.** (DOCX 12 KB)

Additional file 12: **Synteny table of** ***Hbs-1*** **in** ***Medicago truncatula*** **chromosome 5.** (DOCX 12 KB)

Additional file 13: **Synteny of** ***Hbs-1*** **with** ***P. vulgaris.*** Synteny was examined for the *Hbs-1* locus between cowpea and *P.vulgaris* using EST-derived SNP markers previously BLASTed and aligned to the sequenced genome. The *Hbs-1* locus was translated from the cowpea consensus genetic map vs.4 (45.27 cM to 47.18 cM) to vs.6 (49.9 cM to 51.5 cM) on linkage group 5, which corresponded to Phvul.008G213300.1 locus to Phvul.008G214300.1 locus. An ethylene-forming enzyme and an ACC oxidase gene were observed in the region and were considered candidate genes for the *Hbs-1* locus. (TIFF 845 KB)

Additional file 14: **Synteny table of** ***Hbs-1*** **in** ***P. vulgaris*** **chromosome 8.** (DOCX 11 KB)

Additional file 15: **Heat-induced browning of seed coats phenotype.** Cowpea genotypes which are positive for the *Hbs* trait manifest a brown discoloration either partially or over the entire surface of the seed coat when exposed to high temperature heat during flowering. A. RIL number 9 from the IT93K-503-1 x CB46 population which is positive for the heat-induced browning of seed coats (*Hbs)* trait is shown. B. RIL number 8 from the IT93K-503-1 x CB46 population which is negative for the heat-induced browning of seed coats (*hbs*) trait is shown. (TIFF 3 MB)

## Abbreviations

ACC: 1-aminocyclopropane-1 carboxylic acid
ACS: 1-aminocyclopropane-1 carboxylic acid synthase
BAC: Bacterial artificial chromosome
bp: Base pairs
cM: CentiMorgan
DNA J HSP: DNA J heat shock family protein
EFE: Ethylene forming enzyme
ERF: Ethylene responsive element binding factor
EST: Expressed sequence tags
HPR: Hydroxyproline-rich glycoprotein family
HSP: Heat shock proteins
LG: Linkage group
LOD: Logarithm (base 10) of odds
MAS: Marker-assisted selection
Mb: Megabases
QTL: Quantitative trait locus
RIL: Recombinant inbred line
SNP: Single nucleotide polymorphic sequence.
