# Supplementary material for: Identification of candidate genes and molecular markers for heat-induced brown discoloration of seed coats in cowpea [Vigna unguiculata (L.) Walp]
Source: BMC Genomics. 2014 May 1;15(1):328. doi: 10.1186/1471-2164-15-328 (PMC4035059; doi:10.1186/1471-2164-15-328)
Supplement: Supplementary file 1 — Additional file 1: QTL analysis of Hbs-1 in IT93K-503-1 x CB46 population. (DOCX 16 KB) [file 12864_2014_6024_MOESM1_ESM.docx]

| Additional file 1. QTL analysis of *Hbs-1* in the IT93K-503-1 x CB46 population. | | | | | | | |
| --- | --- | --- | --- | --- | --- | --- | --- |
| Experiment | LG | cM | Locus | IM analysis | | Kruskal-Wallis analysis | |
|  |  |  |  | LOD | R^2^ | F-test | p-value |
| F_9_ | 8 | 40.67 | 1_0030 | 3.64 | 15.6 | 16.245 | 0.0001 |
| F_9_ | 8 | 44.29 | 1_0419 | 4.56 | 19.2 | 19.774 | 0.0001 |
| F_9_ | 8 | 46.87 | 1_0242 | 6.15 | 25 | 25.472 | 0.0001 |
| F_9_ | 8 | 47.38 | 1_1419 | 6.15 | 25.1 | 25.472 | 0.0001 |
| F_9_ | 8 | 56.01 | 1_0677 | 11.97 | 43.5 | 43.212 | 0.0001 |
| F_9_ | 8 | 56.01 | 1_1533 | 11.97 | 43.5 | 43.212 | 0.0001 |
| F_9_ | 8 | 58.56 | 1_1322 | 16.48 | 55.6 | 53.946 | 0.0001 |
| F_9_ | 8 | 58.67 | 1_0127 | 16.49 | 55.7 | 53.946 | 0.0001 |
| F_9_ | 8 | 58.67 | 1_0225 | 16.49 | 55.7 | 53.946 | 0.0001 |
| F_9_ | 8 | 58.71 | 1_0081 | 16.5 | 55.7 | 53.946 | 0.0001 |
| F_9_ | 8 | 60.09 | 1_0032 | 19.99 | 62.8 | 60.21 | 0.0001 |
| F_9_ | 8 | 60.53 | 1_1128 | 20.01 | 62.7 | 60.21 | 0.0001 |
| F_9_ | 8 | 64.33 | 1_0226 | 11.63 | 42.1 | 41.119 | 0.0001 |
| F_9_ | 8 | 64.56 | 1_0037 | 11.62 | 42 | 42.613 | 0.0001 |
| F_9_ | 8 | 65.84 | 1_0998 | 9.9 | 36.9 | 37.943 | 0.0001 |
| F_9_ | 8 | 66.04 | 1_0588 | 9.9 | 36.9 | 36.157 | 0.0001 |
| F_9_ | 8 | 68.86 | 1_0379 | 9.89 | 37 | 36.904 | 0.0001 |
| F_9_ | 8 | 71.44 | 1_0579 | 7.18 | 28.7 | 27.787 | 0.0001 |
| F_9_ | 8 | 71.81 | 1_0387 | 6.33 | 25.6 | 24.675 | 0.0001 |
| F_9_ | 8 | 72.48 | 1_1401 | 5.67 | 23.3 | 23.063 | 0.0001 |
| F_9_ | 8 | 74.43 | 1_0923 | 5.01 | 21.1 | 20.774 | 0.0001 |
| F_9_ | 8 | 75.86 | 1_0078 | 4.26 | 18.2 | 17.438 | 0.0001 |
| F_9_ | 8 | 76.13 | 1_1130 | 4.22 | 18 | 17.358 | 0.0001 |
| F_10_ | 8 | 35.21 | 1_1492 | 2.27 | 10.1 | 9.808 | 0.005 |
| F_10_ | 8 | 40.67 | 1_0030 | 4.54 | 19.1 | 19.774 | 0.0001 |
| F_10_ | 8 | 44.29 | 1_0419 | 5.61 | 23.1 | 23.649 | 0.0001 |
| F_10_ | 8 | 46.87 | 1_0242 | 7.44 | 29.5 | 29.864 | 0.0001 |
| F_10_ | 8 | 47.38 | 1_1419 | 7.44 | 29.5 | 29.864 | 0.0001 |
| F_10_ | 8 | 56.01 | 1_0677 | 14.25 | 49.5 | 48.822 | 0.0001 |
| F_10_ | 8 | 56.01 | 1_1533 | 14.25 | 49.5 | 48.822 | 0.0001 |
| F_10_ | 8 | 58.56 | 1_1322 | 24.19 | 70 | 66.818 | 0.0001 |
| F_10_ | 8 | 58.67 | 1_0127 | 24.2 | 70 | 66.818 | 0.0001 |
| F_10_ | 8 | 58.67 | 1_0225 | 24.2 | 70 | 66.818 | 0.0001 |
| F_10_ | 8 | 58.71 | 1_0081 | 24.21 | 70 | 66.818 | 0.0001 |
| F_10_ | 8 | 60.09 | 1_0032 | 30.16 | 77.3 | 73.769 | 0.0001 |
| F_10_ | 8 | 60.53 | 1_1128 | 30.19 | 77.3 | 73.769 | 0.0001 |
| F_10_ | 8 | 64.33 | 1_0226 | 16.44 | 54.2 | 52.537 | 0.0001 |
| F_10_ | 8 | 64.56 | 1_0037 | 16.42 | 54.1 | 54.174 | 0.0001 |
| F_10_ | 8 | 65.84 | 1_0998 | 13.94 | 47.8 | 48.822 | 0.0001 |
| F_10_ | 8 | 66.04 | 1_0588 | 13.92 | 47.7 | 46.722 | 0.0001 |
| F_10_ | 8 | 68.86 | 1_0379 | 13.93 | 47.9 | 47.75 | 0.0001 |
| F_10_ | 8 | 71.44 | 1_0579 | 10.28 | 38.5 | 37.219 | 0.0001 |
| F_10_ | 8 | 71.81 | 1_0387 | 9.13 | 34.7 | 33.738 | 0.0001 |
| F_10_ | 8 | 72.48 | 1_1401 | 8.26 | 32 | 31.77 | 0.0001 |
| F_10_ | 8 | 74.43 | 1_0923 | 7.4 | 29.5 | 29.066 | 0.0001 |
| F_10_ | 8 | 75.86 | 1_0078 | 6.47 | 26.5 | 27.391 | 0.0001 |
| F_10_ | 8 | 76.13 | 1_1130 | 6.41 | 26.2 | 27.177 | 0.0001 |
