# Supplementary figures and images for: Identification of candidate genes and molecular markers for heat-induced brown discoloration of seed coats in cowpea [Vigna unguiculata (L.) Walp]
Source: BMC Genomics. 2014 May 1;15(1):328. doi: 10.1186/1471-2164-15-328 (PMC4035059; doi:10.1186/1471-2164-15-328)

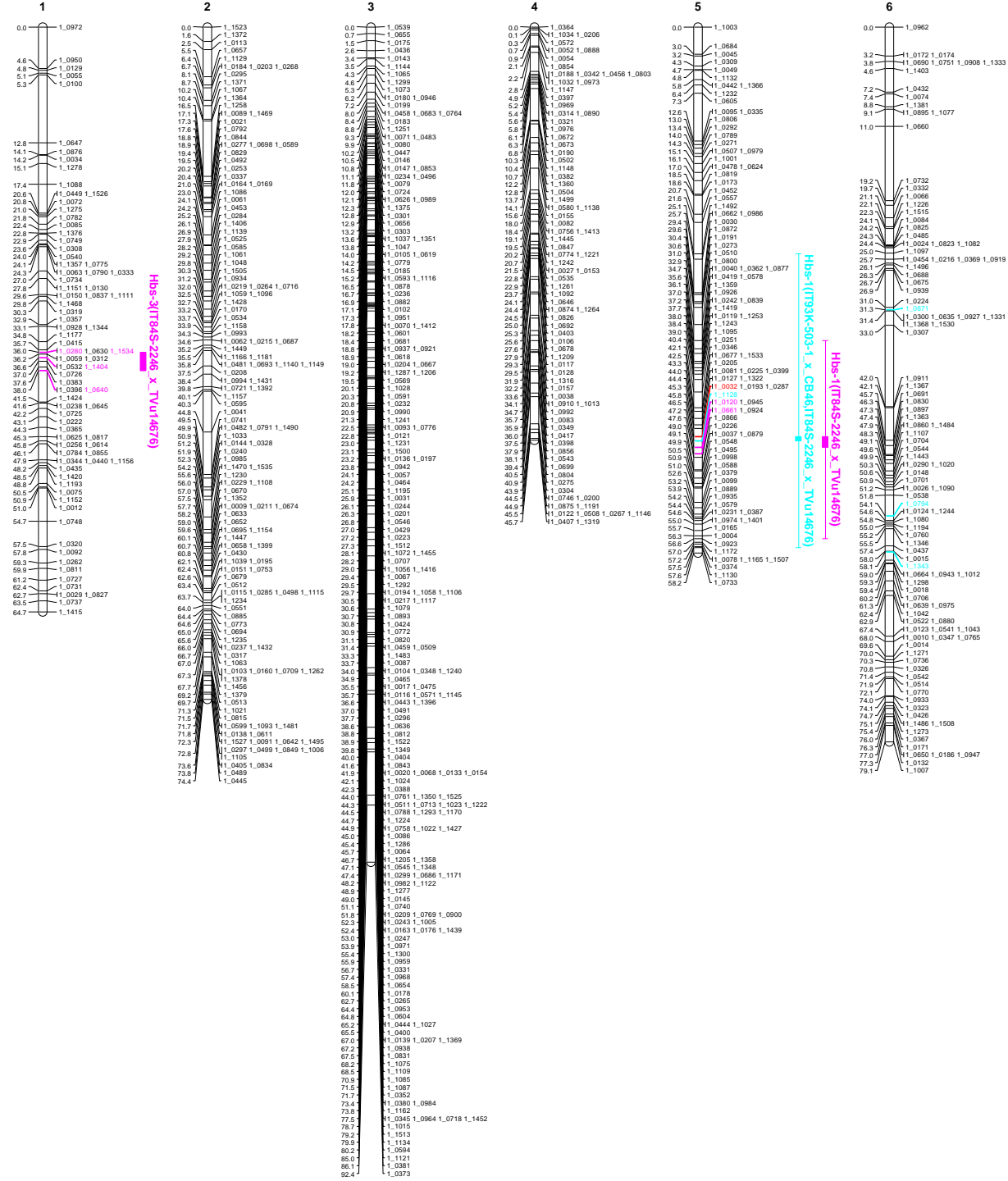

Hbs-2(173K-503-1 x CB46)

Hbs-1(173K-503-1 x CB46)173K-2246 x TV14676

Hbs-1(173K-503-1 x CB46)173K-2246 x TV14676

7

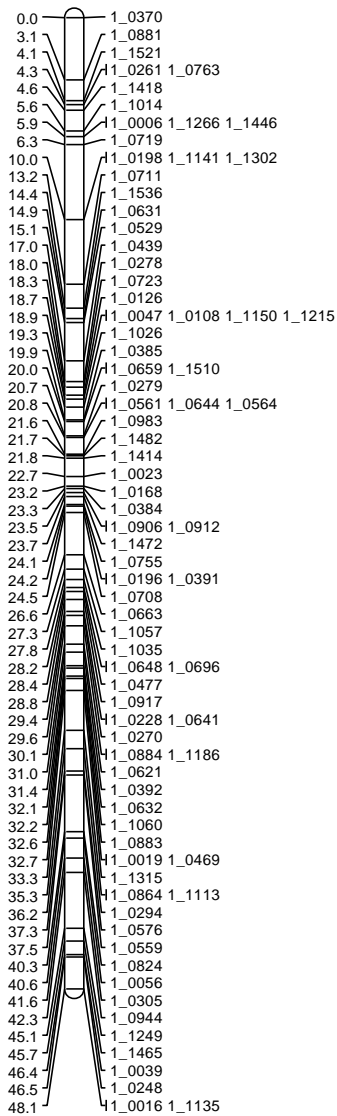

8

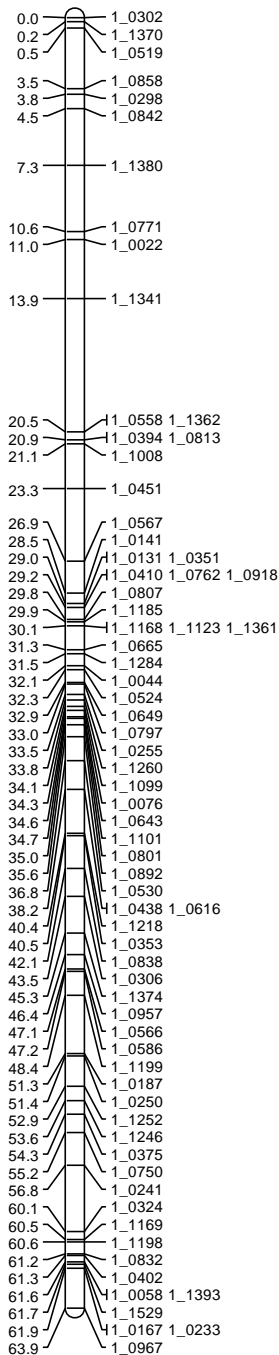

9

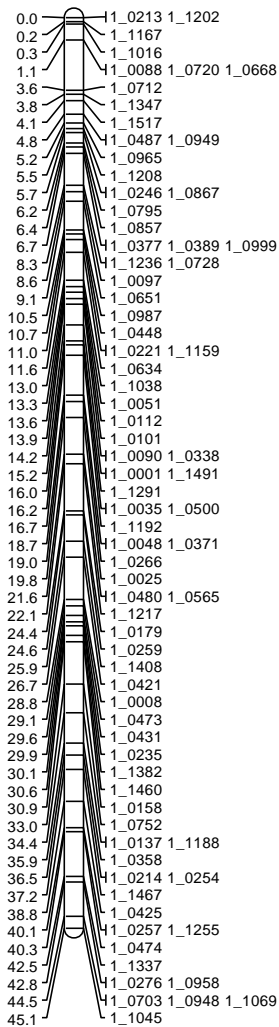

10

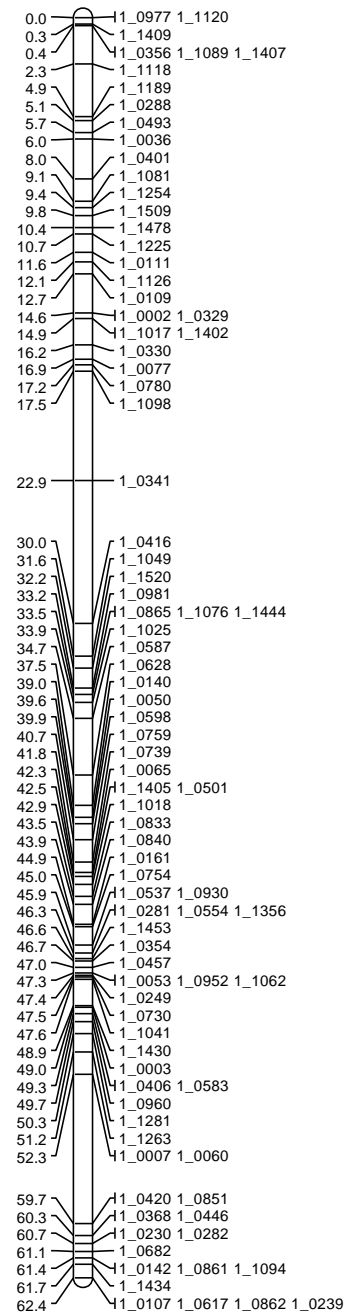

11

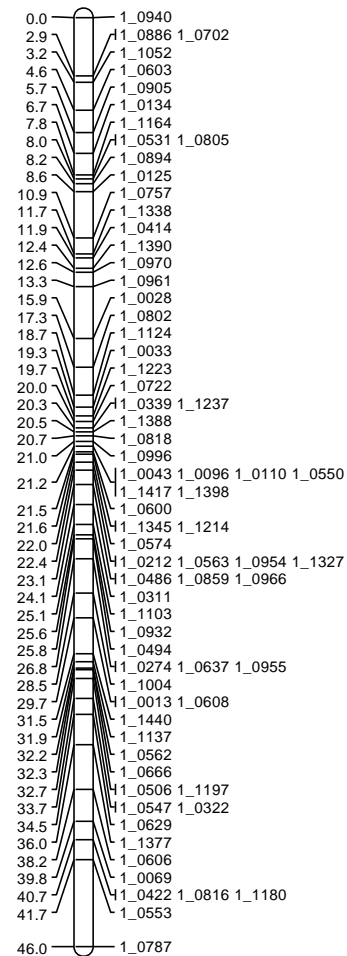

Supplement: Supplementary file 2 — Additional file 2: Hbs-1, Hbs-2 and Hbs-3 on the cowpea consensus genetic map. Heat-induced browning of seed coats QTLs were positioned on the cowpea consensus genetic map using SNP markers identified in the QTL analyses. Hbs-1 and Hbs-2 (labeled light blue) were identified in the IT93K-503-1 x CB46 population. Hbs-1 and Hbs-3 (labeled magenta) were identified in the IT84S-2246 x TVu14676 population. The most significant SNP marker for each QTL is highlighted in the corresponding color on the linkage group. SNP marker 1_0032 is labeled red since it was the most significant marker for both the Hbs-1 locus identified in IT93K-503-1 x CB46 and the IT84S-2246 x TVu14676 population. (PDF 47 KB) [file 12864_2014_6024_MOESM2_ESM.pdf]

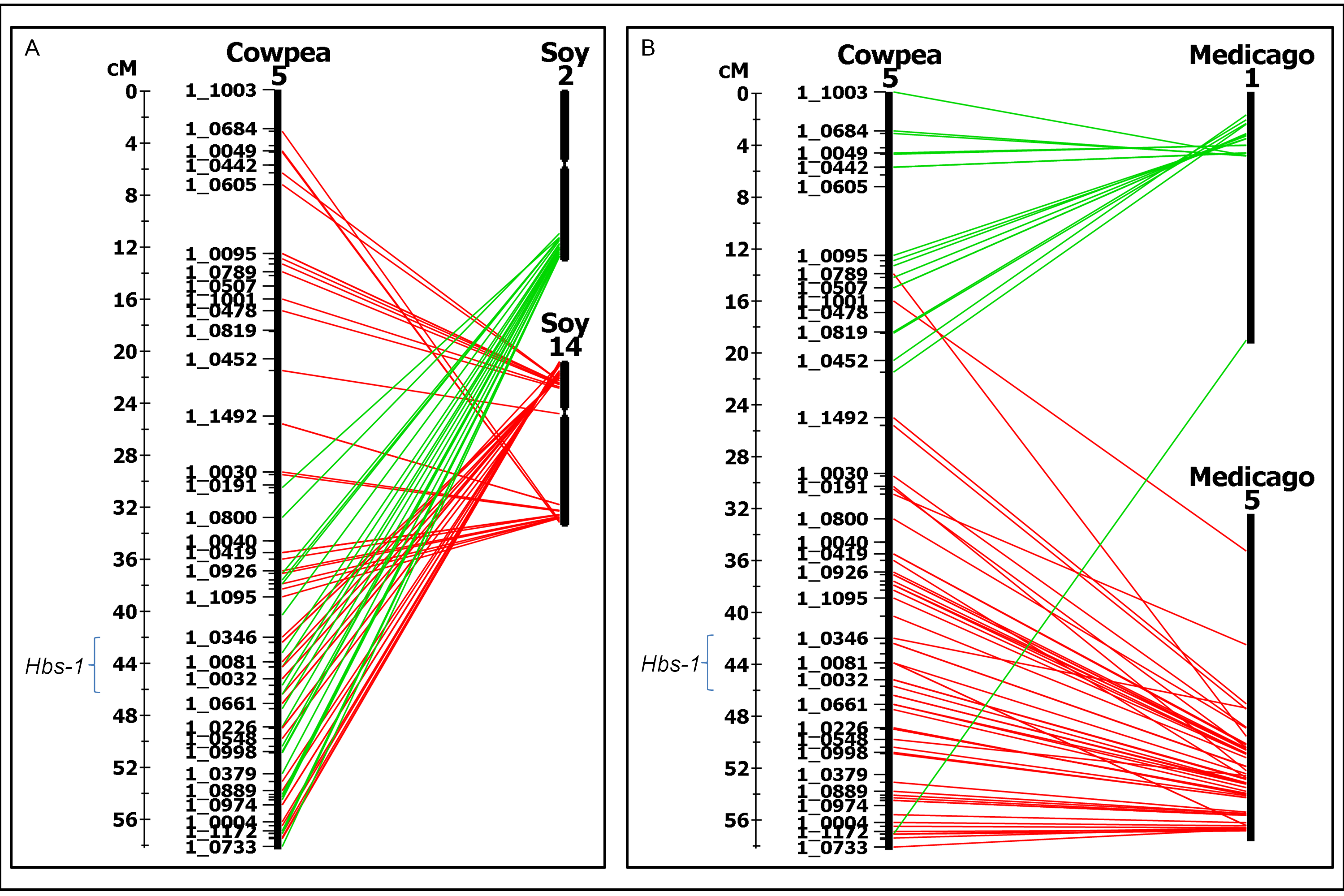

Supplement: Supplementary file 10 — Additional file 10: a. Synteny figure of Hbs-1 locus with G. max. Synteny was examined for the Hbs-1 locus between cowpea and G. max using EST-derived SNP markers previously BLASTed and aligned to the sequenced genome. The Hbs-1 locus which spanned 45.27 cM to 47.18 cM on the cowpea consensus genetic map linkage group 5 was determined to be syntenic with soybean chromosomes 2 and 14. The syntenic locus in soybean chromosome 2 extended from soybean locus Glyma02g42560 to Glyma02g43640 which corresponded to 44.42 cM to 46.51 cM of the Hbs-1 locus. The syntenic locus on soybean chromosome 14 spanned from Glyma14g05250 to Glyma14g06330 which corresponded to 44.42 cM to 47.18 cM of the Hbs-1 locus on the cowpea consensus genetic map. Ethylene responsive element binding factor 3 and 11 and ethylene forming enzymes were observed in the syntenic regions of soybean and were considered candidate genes for the Hbs-1 locus. b. Synteny figure of Hbs-1 locus with M. truncatula. Synteny was examined for the Hbs-1 locus between cowpea and M. truncatula using EST-derived SNP markers previously BLASTed and aligned to the sequenced genome. The Hbs-1 locus which spanned 45.27 cM to 47.18 cM on the cowpea consensus genetic map linkage group 5 was determined to be syntenic with M. truncatula chromosome 5 where it spanned from Medicago locus Medtr5g018870 to Medtr5g093060. Ethylene response factor 3 (ERF3) and an ethylene forming enzyme were present in the locus and were considered candidate genes. (TIFF 2 MB) [file 12864_2014_6024_MOESM10_ESM.tiff]

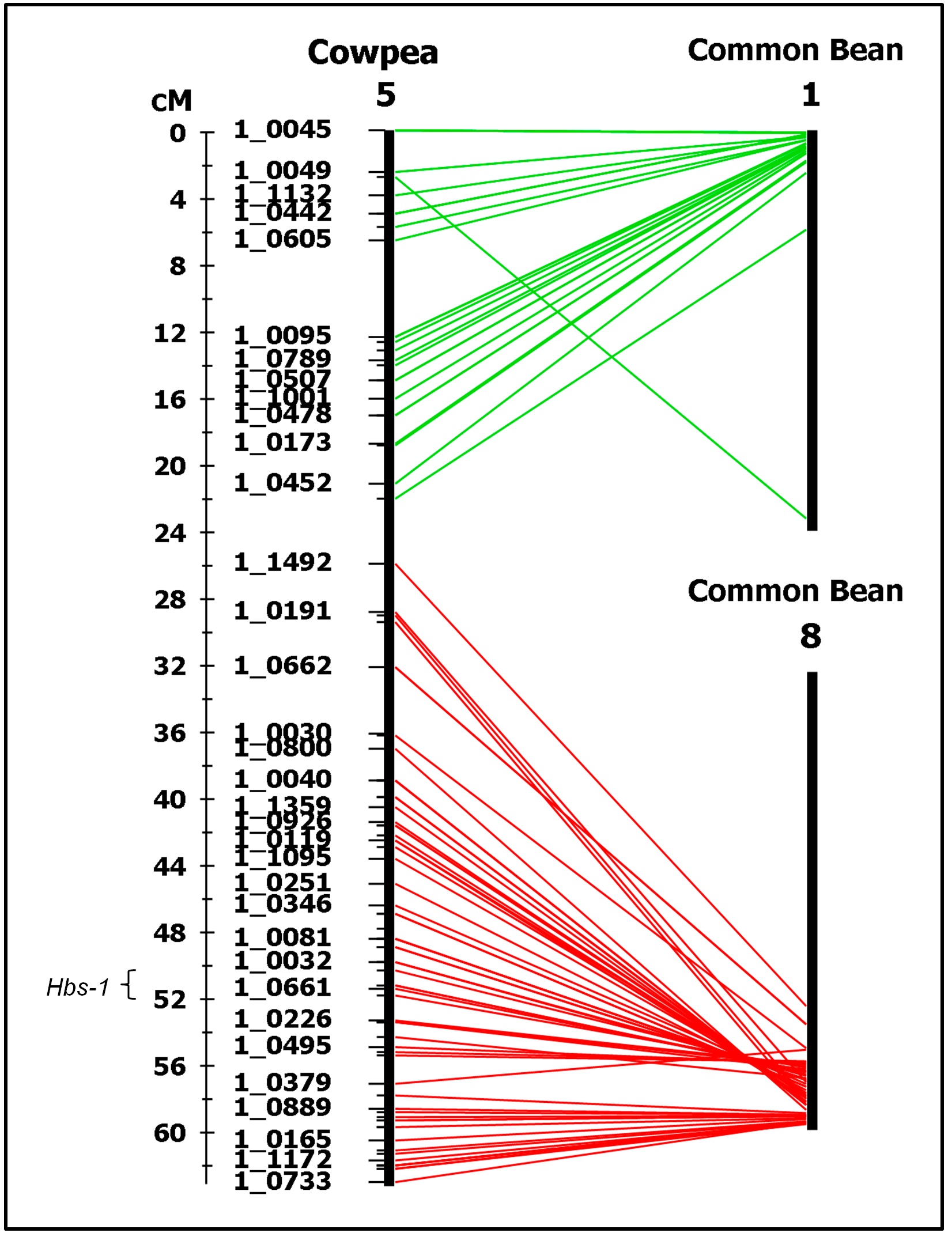

Supplement: Supplementary file 13 — Additional file 13: Synteny of Hbs-1 with P. vulgaris. Synteny was examined for the Hbs-1 locus between cowpea and P.vulgaris using EST-derived SNP markers previously BLASTed and aligned to the sequenced genome. The Hbs-1 locus was translated from the cowpea consensus genetic map vs.4 (45.27 cM to 47.18 cM) to vs.6 (49.9 cM to 51.5 cM) on linkage group 5, which corresponded to Phvul.008G213300.1 locus to Phvul.008G214300.1 locus. An ethylene-forming enzyme and an ACC oxidase gene were observed in the region and were considered candidate genes for the Hbs-1 locus. (TIFF 845 KB) [file 12864_2014_6024_MOESM13_ESM.tiff]

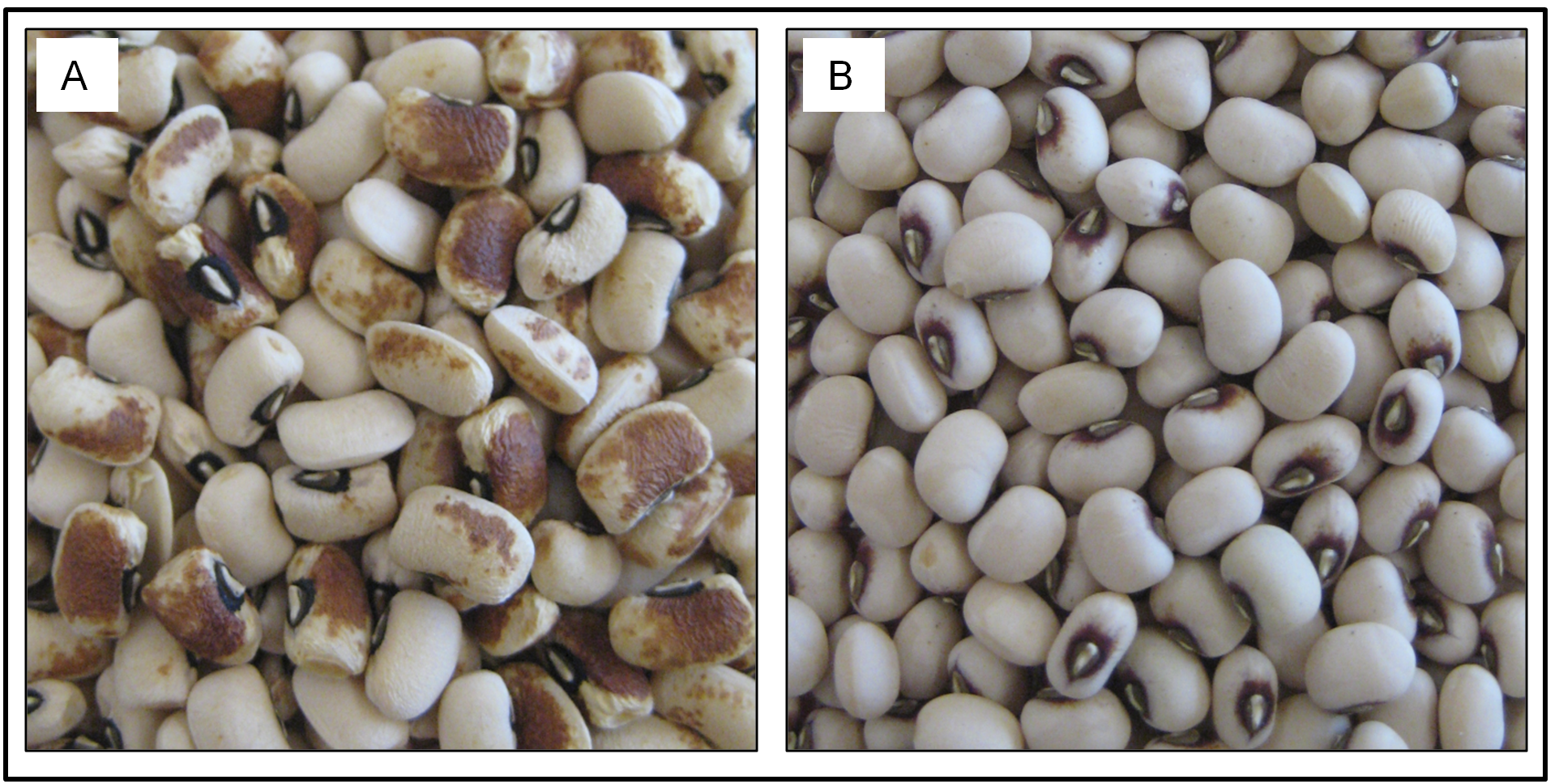

Supplement: Supplementary file 15 — Additional file 15: Heat-induced browning of seed coats phenotype. Cowpea genotypes which are positive for the Hbs trait manifest a brown discoloration either partially or over the entire surface of the seed coat when exposed to high temperature heat during flowering. A. RIL number 9 from the IT93K-503-1 x CB46 population which is positive for the heat-induced browning of seed coats (Hbs) trait is shown. B. RIL number 8 from the IT93K-503-1 x CB46 population which is negative for the heat-induced browning of seed coats (hbs) trait is shown. (TIFF 3 MB) [file 12864_2014_6024_MOESM15_ESM.tiff]
