# Supplementary material for: Identification of candidate genes and molecular markers for heat-induced brown discoloration of seed coats in cowpea [Vigna unguiculata (L.) Walp]
Source: BMC Genomics. 2014 May 1;15(1):328. doi: 10.1186/1471-2164-15-328 (PMC4035059; doi:10.1186/1471-2164-15-328)
Supplement: Supplementary file 3 — Additional file 3: Hbs-1 in IT93K-503-1 x CB46 and IT84S-2246 x TVu14676 individual maps, cowpea consensus genetic map, and the cowpea physical map. (DOCX 14 KB) [file 12864_2014_6024_MOESM3_ESM.docx]

| Additional file 3. *Hbs-1* in IT93K-503-1 x CB46 and IT84S-2246 x TVu14676 individual maps, cowpea consensus genetic map, and the cowpea physical map. | | | | | | | | | | | | | |
| --- | --- | --- | --- | --- | --- | --- | --- | --- | --- | --- | --- | --- | --- |
| IT93K-503-1 x CB46 | | | | IT84S-2246 x TVu14676 | | | | Cowpea consensus genetic map | | | | Cowpea physical map | |
| LG | cM | SNP | LOD | LG | cM | SNP | LOD | LG | cM | SNP | Annotation | contig | BAC(s) |
| 8 | 60.09 | 1_0032 | 30.16 | 9 | 49.51 | 1_0032 | 12.05 | 5 | 45.27 | 1_0032 | SecE/sec61- gamma protein transport protein | N/A | N/A |
|  |  | N/A |  |  | N/A |  |  | 5 | 45.27 | 1_0193 | Ribosomal protein S4 (RPS4A) family protein | N/A | N/A |
|  |  | N/A |  |  | N/A |  |  | 5 | 45.27 | 1_0287 | Glycine-rich protein 3 | N/A | N/A |
| 8 | 60.53 | 1_1128 | 30.19 |  | N/A |  |  | 5 | 45.76 | 1_1128 | Phosphotyrosine protein phosphatase | 217 | CM018C23 |
|  |  | N/A |  | 9 | 50.69 | 1_0120 | 10.41 | 5 | 46.51 | 1_0120 | Ethylene-forming enzyme | 217 | CM018C23 |
|  |  | N/A |  |  |  | N/A |  | 5 | 46.51 | 1_0945 | O-Glycosyl hydrolases family 17 protein | N/A | N/A |
|  |  | N/A |  | 9 | 50.69 | 1_0661 | 10.41 | 5 | 47.18 | 1_0661 | Subtilisin-like serine endopeptidase family protein | N/A | N/A |

Only the most significant region (2-LOD) of *Hbs-1* is shown.
