# Supplementary material for: Identification of candidate genes and molecular markers for heat-induced brown discoloration of seed coats in cowpea [Vigna unguiculata (L.) Walp]
Source: BMC Genomics. 2014 May 1;15(1):328. doi: 10.1186/1471-2164-15-328 (PMC4035059; doi:10.1186/1471-2164-15-328)
Supplement: Supplementary file 4 — Additional file 4: QTL analysis of Hbs-2 in IT93K-503-1 x CB46 population. (DOCX 12 KB) [file 12864_2014_6024_MOESM4_ESM.docx]

| Additional file 4. QTL analysis of *Hbs-2* in the IT93K-503-1 x CB46 population. | | | | | | | |
| --- | --- | --- | --- | --- | --- | --- | --- |
| Experiment | LG | cM | Locus | IM analysis | | Kruskal-Wallis analysis | |
|  |  |  |  | LOD | R^2^ | F-test | p-value |
| F9 | 3 | 50.837 | 1_1343 | 2.77 | 12.3 | 12.3 | 0.0005 |
| F9 | 3 | 51.792 | 1_0871 | 2.61 | 11.7 | 11.7 | 0.001 |
| F10 | 3 | 36.824 | 1_0794 | 2.15 | 9.6 | 9.6 | 0.005 |
| F10 | 3 | 50.837 | 1_1343 | 2.11 | 9.5 | 9.5 | 0.005 |
| F10 | 3 | 51.792 | 1_0871 | 1.98 | 8.9 | 9.15 | 0.005 |
