# Supplementary material for: Identification of candidate genes and molecular markers for heat-induced brown discoloration of seed coats in cowpea [Vigna unguiculata (L.) Walp]
Source: BMC Genomics. 2014 May 1;15(1):328. doi: 10.1186/1471-2164-15-328 (PMC4035059; doi:10.1186/1471-2164-15-328)
Supplement: Supplementary file 5 — Additional file 5: Hbs-2 in IT93K-503-1 x CB46 genetic map, cowpea consensus genetic map, and the cowpea physical map. (DOCX 16 KB) [file 12864_2014_6024_MOESM5_ESM.docx]

| Additional file 5. *Hbs-2* in the IT93K-503-1 x CB46 genetic map, cowpea consensus genetic map and cowpea physical map. | | | | | | | | | |
| --- | --- | --- | --- | --- | --- | --- | --- | --- | --- |
| IT93K-503-1 x CB46 | | | | Cowpea consensus genetic map vs.4 | | | | Cowpea physical map | |
| LG | cM | SNP | LOD | LG | cM | SNP | Annotation | contig | BAC(s) |
| 3 | 51.79 | 1_0871 | 1.98 | 6 | 31.28 | 1_0871 | Transketolase family protein | N/A |  |
|  |  | N/A |  | 6 | 31.42 | 1_0300 | Bifunctional inhibitor/lipid-transfer protein | N/A |  |
|  |  | N/A |  | 6 | 31.42 | 1_0635 | Light harvesting complex photosystem II | N/A |  |
|  |  | N/A |  | 6 | 31.42 | 1_0927 | Tetratricopepti de repeat (TPR)-like protein | 855 | CM018P09 |
|  |  | N/A |  | 6 | 31.42 | 1_1331 | Aldolase-type TIM barrel family protein | N/A |  |
|  |  | N/A |  | 6 | 31.42 | 1_1368 | Methyl-CPG- binding domain 8 | 855 | CM018P09 |
|  |  | N/A |  | 6 | 31.42 | 1_1530 | CD2-binding protein-related | 855 | CM018P09 |
|  |  | N/A |  | 6 | 33.04 | 1_0307 | T-complex protein 11 | 265 | CM005F11, CH084P15 |
|  |  | N/A |  | 6 | 41.95 | 1_0911 | Mitogen-activated protein kinase 16 | 1117 | CM024D19, CM012O18 |
|  |  | N/A |  | 6 | 42.06 | 1_1367 | RNA-binding KH domain-containing protein | 240 | CM013I01 |
|  |  | N/A |  | 6 | 45.75 | 1_0691 | NIFU-like protein 2 | N/A |  |
|  |  | N/A |  | 6 | 46.27 | 1_0830 | Fibrillin | N/A |  |
|  |  | N/A |  | 6 | 47.25 | 1_0897 | Na+/H+ antiporter 6 | 250 | CM015O07, CM002B24 |
|  |  | N/A |  | 6 | 47.41 | 1_1363 | Eukaryotic aspartyl protease family protein | 250 | CM015O07 |
| 3 | 39.59 | 1_0860 | 1.79 | 6 | 47.86 | 1_0860 | Vesicle- associated membrane protein 726 | 250 | CH076D23, CH093L18 |
| 3 | 39.66 | 1_1484 | 1.79 | 6 | 47.93 | 1_1484 | Kinase-related protein of unknown function | 250 | CH076D23, CH093L18 |
| 3 | 39.74 | 1_1107 | 1.58 | 6 | 48.31 | 1_1107 | Chloroplastic NIFS-like cysteine desulfurase | 250 | CH051M10, CM001C09, M019E01 |
|  |  | N/A |  | 6 | 49.14 | 1_0704 | Glycolipid transfer protein | 250 | CH051M10 |
|  |  | N/A |  | 6 | 49.60 | 1_0544 | Peroxidase superfamily protein | N/A |  |
| 3 | 39.82 | 1_1443 | 1.56 | 6 | 49.85 | 1_1443 | Peroxidase superfamily protein | N/A |  |
| 3 | 40.15 | 1_0290 | 1.55 | 6 | 50.34 | 1_0290 | Ribosomal protein L13 family protein | 551 | CM030A23, CM054O22 |
| 3 | 40.15 | 1_1020 | 1.55 | 6 | 50.34 | 1_1020 | Root FNR 1 | 551 | CM030A23, CM054O22 |
| 3 | 39.23 | 1_0148 | 1.57 | 6 | 50.57 | 1_0148 | GTP binding | 551 | CM054022, CM030A23 |
| 3 | 40.15 | 1_0701 | 1.55 | 6 | 50.92 | 1_0701 | Putative type 1 membrane protein | N/A |  |
| 3 | 40.42 | 1_0026 | 1.55 | 6 | 51.21 | 1_0026 | Tubulin beta 8 | 691 | CH024L08 |
|  |  | N/A |  | 6 | 51.21 | 1_1090 | 6- phosphogluconate dehydrogenase | 551 | CM030A23 |
|  |  | N/A |  | 6 | 51.81 | 1_0538 | fatty acid hydroxylase 1 | 691 | CH071M11 |
| 3 | 36.82 | 1_0794 | 2.15 | 6 | 54.05 | 1_0794 | Rab5- interacting family protein | N/A |  |
|  |  | N/A |  | 6 | 54.61 | 1_0124 | ATP-citrate lyase B-1 | N/A |  |
|  |  | N/A |  | 6 | 54.61 | 1_1244 | DNA-binding enhancer protein-related | 38 | CH074G16 |
|  |  | N/A |  | 6 | 54.78 | 1_1080 | Phytochrome B | N/A |  |
|  |  | N/A |  | 6 | 54.99 | 1_1194 | No functional annotation | N/A |  |
|  |  | N/A |  | 6 | 55.25 | 1_0760 | ATPase, F1 complex | N/A |  |
|  |  | N/A |  | 6 | 55.50 | 1_1346 | Ubiquitin-like superfamily protein | N/A |  |
|  |  | N/A |  | 6 | 57.41 | 1_0437 | Bacterial sec-independent translocation protein | 240 | CH077I19, CM023J02 |
|  |  | N/A |  | 6 | 57.97 | 1_0015 | Acyl carrier protein 4 | 707 | CM054P06 |
| 3 | 50.84 | 1_1343 | 2.11 | 6 | 58.09 | 1_1343 | Ribosomal protein L18ae/LX family protein | 606 | CH082B14 |
