# Supplementary material for: Identification of candidate genes and molecular markers for heat-induced brown discoloration of seed coats in cowpea [Vigna unguiculata (L.) Walp]
Source: BMC Genomics. 2014 May 1;15(1):328. doi: 10.1186/1471-2164-15-328 (PMC4035059; doi:10.1186/1471-2164-15-328)
Supplement: Supplementary file 6 — Additional file 6: QTL analysis of Hbs-1 in the IT84S-2246 x TVu14676 population. (DOCX 16 KB) [file 12864_2014_6024_MOESM6_ESM.docx]

| Additional file 6. QTL mapping of *Hbs-1* in the IT84S-2246 x TVu14676 population. | | | | | | | |
| --- | --- | --- | --- | --- | --- | --- | --- |
| Experiment | LG | cM | Locus | IM analysis | | Kruskal-Wallis analysis | |
|  |  |  |  | LOD | R^2^ | F-test | p-value |
| F9 | 9 | 24.98 | 1_1492 | 2.39 | 7.9 | 10.171 | 0.005 |
| F9 | 9 | 30.84 | 1_0800 | 1.83 | 6.2 | 8.121 | 0.005 |
| F9 | 9 | 33.77 | 1_0877 | 2.09 | 7 | 9.199 | 0.005 |
| F9 | 9 | 33.77 | 1_0362 | 2.09 | 7 | 9.199 | 0.005 |
| F9 | 9 | 33.77 | 1_0040 | 2.09 | 7 | 9.199 | 0.005 |
| F9 | 9 | 34.63 | 1_1359 | 2.24 | 7.4 | 9.832 | 0.005 |
| F9 | 9 | 40.41 | 1_0119 | 3.83 | 12.4 | 16.381 | 0.0001 |
| F9 | 9 | 40.41 | 1_1243 | 3.83 | 12.4 | 16.381 | 0.0001 |
| F9 | 9 | 41.23 | 1_0251 | 3.83 | 12.4 | 16.381 | 0.0001 |
| F9 | 9 | 45.29 | 1_1533 | 6.34 | 19.7 | 26.002 | 0.0001 |
| F9 | 9 | 45.29 | 1_0677 | 6.34 | 19.7 | 26.002 | 0.0001 |
| F9 | 9 | 47.48 | 1_0225 | 8.95 | 26.6 | 35.178 | 0.0001 |
| F9 | 9 | 47.48 | 1_0081 | 8.95 | 26.6 | 35.178 | 0.0001 |
| F9 | 9 | 49.51 | 1_0032 | 12.05 | 34.1 | 45.032 | 0.0001 |
| F9 | 9 | 50.69 | 1_0661 | 10.41 | 30.3 | 39.953 | 0.0001 |
| F9 | 9 | 50.69 | 1_0120 | 10.41 | 30.3 | 39.953 | 0.0001 |
| F9 | 9 | 51.67 | 1_0226 | 9.36 | 27.7 | 36.523 | 0.0001 |
| F9 | 9 | 51.67 | 1_0037 | 9.36 | 27.7 | 36.523 | 0.0001 |
| F9 | 9 | 51.94 | 1_0495 | 9.36 | 27.7 | 36.523 | 0.0001 |
| F9 | 9 | 52.16 | 1_0998 | 9.78 | 28.7 | 37.915 | 0.0001 |
| F9 | 9 | 52.28 | 1_0588 | 9.78 | 28.7 | 37.915 | 0.0001 |
| F9 | 9 | 53.78 | 1_0379 | 6.8 | 21 | 27.7 | 0.0001 |
| F9 | 9 | 57.07 | 1_0974 | 5.34 | 16.9 | 22.268 | 0.0001 |
| F9 | 9 | 57.07 | 1_1401 | 5.34 | 16.9 | 22.268 | 0.0001 |
| F9 | 9 | 57.82 | 1_0387 | 5.34 | 16.9 | 22.268 | 0.0001 |
| F9 | 9 | 57.82 | 1_0579 | 5.34 | 16.9 | 22.268 | 0.0001 |
| F9 | 9 | 59.60 | 1_0923 | 4.52 | 14.5 | 19.116 | 0.0001 |
| F10 | 9 | 24.98 | 1_1492 | 3.81 | 12.4 | 15.896 | 0.0001 |
| F10 | 9 | 30.84 | 1_0800 | 2.45 | 8.2 | 10.741 | 0.005 |
| F10 | 9 | 33.77 | 1_0877 | 2.71 | 9 | 11.829 | 0.001 |
| F10 | 9 | 33.77 | 1_0362 | 2.71 | 9 | 11.829 | 0.001 |
| F10 | 9 | 33.77 | 1_0040 | 2.71 | 9 | 11.829 | 0.001 |
| F10 | 9 | 34.63 | 1_1359 | 2.9 | 9.6 | 12.591 | 0.0005 |
| F10 | 9 | 40.41 | 1_0119 | 3.94 | 12.9 | 16.841 | 0.0001 |
| F10 | 9 | 40.41 | 1_1243 | 3.94 | 12.9 | 16.841 | 0.0001 |
| F10 | 9 | 41.23 | 1_0251 | 3.94 | 12.9 | 16.841 | 0.0001 |
| F10 | 9 | 45.29 | 1_1533 | 5.56 | 17.6 | 23.08 | 0.0001 |
| F10 | 9 | 45.29 | 1_0677 | 5.56 | 17.6 | 23.08 | 0.0001 |
| F10 | 9 | 47.48 | 1_0225 | 7.81 | 23.9 | 31.257 | 0.0001 |
| F10 | 9 | 47.48 | 1_0081 | 7.81 | 23.9 | 31.257 | 0.0001 |
| F10 | 9 | 49.51 | 1_0032 | 9.54 | 28.3 | 37.093 | 0.0001 |
| F10 | 9 | 50.69 | 1_0661 | 8.2 | 24.9 | 32.595 | 0.0001 |
| F10 | 9 | 50.69 | 1_0120 | 8.2 | 24.9 | 32.595 | 0.0001 |
| F10 | 9 | 51.67 | 1_0226 | 7.36 | 22.7 | 29.676 | 0.0001 |
| F10 | 9 | 51.67 | 1_0037 | 7.36 | 22.7 | 29.676 | 0.0001 |
| F10 | 9 | 51.94 | 1_0495 | 7.36 | 22.7 | 29.676 | 0.0001 |
| F10 | 9 | 52.16 | 1_0998 | 7.74 | 23.7 | 31.01 | 0.0001 |
| F10 | 9 | 52.28 | 1_0588 | 7.74 | 23.7 | 31.01 | 0.0001 |
| F10 | 9 | 53.78 | 1_0379 | 5.25 | 16.7 | 21.942 | 0.0001 |
| F10 | 9 | 57.07 | 1_0974 | 5.42 | 17.2 | 22.574 | 0.0001 |
| F10 | 9 | 57.07 | 1_1401 | 5.42 | 17.2 | 22.574 | 0.0001 |
| F10 | 9 | 57.82 | 1_0387 | 5.42 | 17.2 | 22.574 | 0.0001 |
| F10 | 9 | 57.82 | 1_0579 | 5.42 | 17.2 | 22.574 | 0.0001 |
| F10 | 9 | 59.60 | 1_0923 | 4.69 | 15.1 | 19.788 | 0.0001 |
