# Supplementary material for: Identification of candidate genes and molecular markers for heat-induced brown discoloration of seed coats in cowpea [Vigna unguiculata (L.) Walp]
Source: BMC Genomics. 2014 May 1;15(1):328. doi: 10.1186/1471-2164-15-328 (PMC4035059; doi:10.1186/1471-2164-15-328)
Supplement: Supplementary file 7 — Additional file 7: QTL analysis of Hbs-3 in the IT84S-2246 x TVu14676 population. (DOCX 12 KB) [file 12864_2014_6024_MOESM7_ESM.docx]

| Additional file 7. QTL analysis of *Hbs-3* in the IT84S-2246 x TVu14676 population. | | | | | | | |
| --- | --- | --- | --- | --- | --- | --- | --- |
| Experiment | LG | cM | SNP marker | IM analysis | | Kruskal-Wallis analysis | |
|  |  |  |  | LOD | R^2^ | F-test | p-value |
| F_9_ | 3 | 17.79 | 1_0280 | 1.85 | 6.2 | 8.193 | 0.005 |
| F_9_ | 3 | 17.79 | 1_1534 | 1.85 | 6.2 | 8.193 | 0.005 |
| F_9_ | 3 | 17.79 | 1_1404 | 1.85 | 6.2 | 8.193 | 0.005 |
| F_9_ | 3 | 20.97 | 1_0640 | 0.71 | 2.4 | 3.066 | 0.1 |
| F_10_ | 3 | 17.79 | 1_0280 | 2.02 | 6.8 | 8.925 | 0.005 |
| F_10_ | 3 | 17.79 | 1_1534 | 2.02 | 6.8 | 8.925 | 0.005 |
| F_10_ | 3 | 17.79 | 1_1404 | 2.02 | 6.8 | 8.925 | 0.005 |
| F_10_ | 3 | 20.97 | 1_0640 | 0.84 | 2.9 | 3.625 | 0.1 |
