# Supplementary material for: Identification of candidate genes and molecular markers for heat-induced brown discoloration of seed coats in cowpea [Vigna unguiculata (L.) Walp]
Source: BMC Genomics. 2014 May 1;15(1):328. doi: 10.1186/1471-2164-15-328 (PMC4035059; doi:10.1186/1471-2164-15-328)
Supplement: Supplementary file 8 — Additional file 8: Hbs-3 in the IT84S-2246 x TVu14676 population, cowpea consensus genetic map and cowpea physical map. (DOCX 14 KB) [file 12864_2014_6024_MOESM8_ESM.docx]

| Additional file 8. *Hbs-3* in IT84S-2246 x TVu14676 individual map, cowpea consensus genetic map, and the cowpea physical map. | | | | | | | | | |
| --- | --- | --- | --- | --- | --- | --- | --- | --- | --- |
| IT84S-2246 x TVu14676 | | | | Cowpea consensus genetic map vs.4 | | | | Cowpea physical map | |
| LG | cM | SNP | LOD | LG | cM | SNP | Annotation | contig | BAC(s) |
| 3 | 17.79 | 1_0280 | 2.02 | 1 | 36.00 | 1_0280 | Mitochondrial acyl carrier protein 2 | 674 | CM059B13 |
|  |  | N/A |  | 1 | 36.02 | 1_0630 | Thioredoxin F2 | N/A | N/A |
| 3 | 17.79 | 1_1534 | 2.02 | 1 | 36.02 | 1_1534 | High chlorophyll fluorescent 109 | 674 | CM059B13, CH093M18 |
|  |  | N/A |  | 1 | 36.25 | 1_0059 | No functional annotation | 674 | CM013N21 |
|  |  | N/A |  | 1 | 36.25 | 1_0312 | 2-phosphoglycolate phosphatase 1 | 674 | CM013N21 |
|  |  | N/A |  | 1 | 36.56 | 1_0532 | Chloroplastic acetylcoenzyme A carboxylase 1 | 674 | CM059B13, CH093M18 |
| 3 | 17.79 | 1_1404 | 2.02 | 1 | 36.56 | 1_1404 | No functional annotation | N/A | N/A |
|  |  | N/A |  | 1 | 36.99 | 1_0726 | Uncharacterized protein family (UPF0016) | N/A | N/A |
|  |  | N/A |  | 1 | 37.64 | 1_0383 | Ribosomal L5P family protein | 512 | CM042F21, CM014K16 |
|  |  | N/A |  | 1 | 37.96 | 1_0396 | No functional annotation | 512 | CM042F21 |
| 3 | 20.97 | 1_0640 | 0.84 | 1 | 37.96 | 1_0640 | Ubiquitin- conjugating enzyme 5 | N/A | N/A |
