# Supplementary material for: Identification of candidate genes and molecular markers for heat-induced brown discoloration of seed coats in cowpea [Vigna unguiculata (L.) Walp]
Source: BMC Genomics. 2014 May 1;15(1):328. doi: 10.1186/1471-2164-15-328 (PMC4035059; doi:10.1186/1471-2164-15-328)
Supplement: Supplementary file 9 — Additional file 9: Origins of genotypes used for the marker-trait association. (DOCX 12 KB) [file 12864_2014_6024_MOESM9_ESM.docx]

| Additional file 9. Origins of genotypes used for the marker-trait association in the *Hbs-1* and *Hbs-3* loci. | | | | | | | |
| --- | --- | --- | --- | --- | --- | --- | --- |
| Cultivar | Reference | Country of origin | Type | *Hbs* phenotype | *Hbs-1* 1_0032 (45.27 cM/LG5) | *Hbs-1* 1_1128  (45.76 cM/LG5) | *Hbs-2* 1_0640 (37.96 cM/LG1) |
| IT84S-2246 (PI 582519) |  | Nigeria | IITA breeding line | Positive | AA | TT | AA |
| IT93K-503-1 |  | Nigeria | IITA breeding line | Positive | AA | TT | AA |
| IT93K-2046 |  | N/A | IITA breeding line | Positive | AA | TT | AA |
| TVu-4552 | Hall and Patel (1985) | Nigeria |  | Positive | AA | TT | AA |
| TVx-3236 (PI 632845) | Hall and Patel (1985) | N/A | IITA breeding line | Positive | AA | TT | AA |
| TVu-53 |  | Nigeria |  | Positive | AA | TT | AA |
| TVu-15315 |  | Chad |  | Positive | AA | TT | AA |
| TVu-14676 |  | Botswana | Traditional cultivar/Landrace | Negative | GG | AA | GG |
| CB5 | Hall and Patel (1985) | U.S.A. | Improved variety | Negative | GG | AA | GG |
| CB27 |  | U.S.A. | Improved variety | Negative | GG | AA | GG |
| CB46 |  | U.S.A. | Improved variety | Negative | GG | AA | GG |
| 524B |  | U.S.A. | Improved cultivar (CB5 x CB3) | Negative | GG | AA | GG |
| Bambey 21 | Hall and Patel (1985) | Senegal | Improved variety | Negative | GG | AA | GG |
