# Supplementary material for: Identification of candidate genes and molecular markers for heat-induced brown discoloration of seed coats in cowpea [Vigna unguiculata (L.) Walp]
Source: BMC Genomics. 2014 May 1;15(1):328. doi: 10.1186/1471-2164-15-328 (PMC4035059; doi:10.1186/1471-2164-15-328)
Supplement: Supplementary file 11 — Additional file 11: Synteny table of Hbs-1 in Glycine max chromosomes 2 and 14. (DOCX 12 KB) [file 12864_2014_6024_MOESM11_ESM.docx]

| Additional file 11. Synteny of *Hbs-1* in *Glycine max* chromosomes 2 and 14*.* | | | | | |
| --- | --- | --- | --- | --- | --- |
| *G. max* chromosome | *G. max* locus | Phytozome annotation | Cowpea locus | cM | LG |
| Gm02 | Glyma02g42560 | Vesicle coat protein clathrin, heavy chain | 1_0127 | 44.42 | 5 |
| Gm02 | Glyma02g43500 | ATERF3/ERF3 (Ethylene responsive element binding factor 3) | N/A | N/A | N/A |
| Gm02 | Glyma02g43550 | Tyrosine phosphatase family | 1_1128 | 45.76 | 5 |
| Gm02 | Glyma02g43560 | EFE (Ethylene forming enzyme) | 1_0120 | 46.51 | 5 |
| Gm02 | Glyma02g43580 | EFE (Ethylene forming enzyme) | N/A | N/A | N/A |
| Gm02 | Glyma02g43600 | EFE (Ethylene forming enzyme) | N/A | N/A | N/A |
| Gm02 | Glyma02g43640 | Glycosyl hydrolases family | 1_0945 | 46.51 | 5 |
| Gm14 | Glyma14g05250 | Subtilase family | 1_0661 | 47.18 | 5 |
| Gm14 | Glyma14g05270 | Subtilase family | 1_0661 | 47.18 | 5 |
| Gm14 | Glyma14g05300 | Glycosyl hydrolases family | 1_0945 | 46.51 | 5 |
| Gm14 | Glyma14g05350 | EFE (Ethylene forming enzyme) | N/A | N/A | N/A |
| Gm14 | Glyma14g05360 | EFE (Ethylene forming enzyme) | N/A | N/A | N/A |
| Gm14 | Glyma14g05390 | EFE (Ethylene forming enzyme) | 1_0120 | 46.51 | 5 |
| Gm14 | Glyma14g05400 | Tyrosine phosphatase family | 1_1128 | 45.76 | 5 |
| Gm14 | Glyma14g05470 | ATERF3/ERF3 (Ethylene responsive element binding factor 3) | N/A | N/A | N/A |
| Gm14 | Glyma14g05800 | SecE/Sec61-gamma subunits | 1_0032 | 45.27 | 5 |
| Gm14 | Glyma14g06330 | CIRCADIAN PROTEIN CLOCK/ARNT/BMAL/PAS | 1_1322 | 44.42 | 5 |
