# Supplementary material for: Identification of candidate genes and molecular markers for heat-induced brown discoloration of seed coats in cowpea [Vigna unguiculata (L.) Walp]
Source: BMC Genomics. 2014 May 1;15(1):328. doi: 10.1186/1471-2164-15-328 (PMC4035059; doi:10.1186/1471-2164-15-328)
Supplement: Supplementary file 12 — Additional file 12: Synteny table of Hbs-1 in Medicago truncatula chromosome 5. (DOCX 12 KB) [file 12864_2014_6024_MOESM12_ESM.docx]

| Additional file 12. Synteny of *Hbs-1* in *Medicago truncatula* chromosome 5. | | | | | |
| --- | --- | --- | --- | --- | --- |
| *M. truncatula* chromosome | *M. truncatula* locus | Phytozome annotation | Cowpea locus | cM | LG |
| 5 | Medtr5g018870 | 40S ribosomal protein S4 (RPS4B) | 45.27 | 1_0193 | 5 |
| 5 | Medtr5g090360 | Clathrin, heavy-chain linker | 44.42 | 1_0127 | 5 |
| 5 | Medtr5g091490 | Protein transport protein SEC61 gamma subunit | 45.27 | 1_0032 | 5 |
| 5 | Medtr5g091750 | Glycine-rich protein | 45.27 | 1_0287 | 5 |
| 5 | Medtr5g091880 | Glycine-rich protein | 45.27 | 1_0287 | 5 |
| 5 | Medtr5g092410 | ERF3 (Ethylene responsive element binding factor 3) | N/A |  |  |
| 5 | Medtr5g092450 | ERF3 (Ethylene responsive element binding factor 3) | N/A |  |  |
| 5 | Medtr5g092470 | ERF3 (Ethylene responsive element binding factor 3) | N/A |  |  |
| 5 | Medtr5g092480 | ERF11 (ERF DOMAIN PROTEIN 11) | N/A |  |  |
| 5 | Medtr5g092680 | Tyrosine specific protein phosphatase family protein | 45.76 | 1_1128 | 5 |
| 5 | Medtr5g092760 | EFE (Ethylene forming enzyme) | 46.51 | 1_0120 | 5 |
| 5 | Medtr5g093030 | Glycosyl hydrolases family | 46.51 | 1_0945 | 5 |
| 5 | Medtr5g093060 | Subtilisin/kexin-related serine protease | 47.18 | 1_0661 | 5 |
