# Supplementary material for: Identification of candidate genes and molecular markers for heat-induced brown discoloration of seed coats in cowpea [Vigna unguiculata (L.) Walp]
Source: BMC Genomics. 2014 May 1;15(1):328. doi: 10.1186/1471-2164-15-328 (PMC4035059; doi:10.1186/1471-2164-15-328)
Supplement: Supplementary file 14 — Additional file 14: Synteny table of Hbs-1 in P. vulgaris chromosome 8. (DOCX 11 KB) [file 12864_2014_6024_MOESM14_ESM.docx]

| Additional file 14. Synteny of *Hbs-1* in *P. vulgaris* chromosome 8*.* | | | | | |
| --- | --- | --- | --- | --- | --- |
| *P. vulgaris* chromosome | *P. vulgaris* locus | *P. vulgaris* annotation | Cowpea SNP | LG | cM |
| 8 | Phvul.008G213300.1 | Subtilisin-like serine endopeptidase family protein | 1_0661 | 5 | 47.18 |
| 8 | Phvul.008G213400.1 | O-Glycosyl hydrolases family 17 protein | 1_0945 | 5 | 46.51 |
| 8 | Phvul.008G213800.1 | Ethylene-forming enzyme | N/A |  |  |
| 8 | Phvul.008G213900.1 | ACC oxidase 2 | N/A |  |  |
| 8 | Phvul.008G214200.1 | Ethylene-forming enzyme | 1_0120 | 5 | 46.51 |
| 8 | Phvul.008G214300.1 | Phosphotyrosine protein phosphatases superfamily protein | 1_1128 | 5 | 45.76 |
